# Representative volume elements of strain/stress fields measured by diffraction techniques

**DOI:** 10.1107/S1600576723004351

**Published:** 2023-07-20

**Authors:** Mehmet Hazar Şeren, Darren C. Pagan, Ismail Cevdet Noyan

**Affiliations:** a Department of Applied Physics and Applied Mathematics, SEAS, Columbia University, 500W 120th Street, New York, NY 10027, USA; b ASML (United States) LP, 77 Danbury Road, Wilton, CT 06897, USA; c Materials Science and Engineering and Mechanical Engineering, Pennsylvania State University, 328 Steidle Building, University Park, PA 16802, USA; Montanuniversität Leoben, Austria

**Keywords:** diffraction analysis, stress, strain, polycrystalline solids

## Abstract

Finite-element modelling has been used to simulate local strains and stresses within free-standing polycrystalline slabs of W, Cu and W–Cu, heated with free or constrained boundaries. The elastic strain values in crystallites that satisfied the diffraction condition were used to simulate the lattice strain data that would be obtained from diffraction analysis, from which the average stresses within diffracting domains were computed. Comparison of direct-space stresses in the model with the average stresses determined from diffraction analysis shows that the representative volume elements (RVEs) required to obtain equivalent stress/strain values depend on the deformation mode suffered by the material. Further, the direct-space and diffraction stress values agree only under strict sampling and strain/stress uniformity conditions. Consequently, in samples where measurements are conducted in volumes smaller than the RVE, or where the uniformity conditions are not satisfied, further experimental and numerical techniques might be needed for the accurate determination of applied or residual stress distributions.

## Abstract 2

Representative volume elements for diffraction-based stress/strain distributions in polycrystalline materials are compared with direct-space values of these quantities using numerical modelling. The results indicate that these volumes and their stress states are equivalent under very specific conditions.

## 1. Nomenclature

**σ** ^0^: uniform far-field stress field applied at the sample boundary.

**ε** ^0^: (homogeneous) strain matrix induced in an equivalent isotropic sample in response to **σ** ^0^.

*V* ^0^: total sample volume.

〈**σ**〉_*V*_: average stress over arbitrary volume *V*.


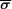
 : average stress for the entire sample volume *V* ^0^.


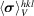
 : average stress for all grains within volume *V* diffracting into reflection *hkl*.

**σ**(*Q*), **ε**(*Q*): local stress and strain matrices, respectively, at point *Q*.

**C**(*Q*), **S**(*Q*): local stiffness and compliance matrices, respectively, at point *Q*.


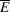
 , 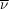 : average Young modulus and Poisson ratio, respectively, of the sample volume.

RLE, RAE, RVE: representative line, area and volume elements, respectively.

RVE_DS_: direct-space representative volume element.


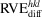
 : diffraction representative volume element.

**P**: sample coordinate system.

**L**: laboratory coordinate system.

*a*_*uv*_: components of the direction cosine matrix linking **P** and **L**.

σ*(*Q*), ε*(*Q*): interaction stress and strain matrices, respectively, at point *Q* (Heyn stresses and strains).

MS: meso-scale.

σ**(*Q*), ε**(*Q*): Saint-Venant stresses and strains, respectively, at point *Q*.

**q**_*hkl*_: unit scattering vector normal to the diffracting planes. **q**_*hkl*_ || 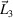 .


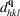
 : atomic plane spacing of a particular family of planes [*hkl*] along the specific scattering vector **q**_*hkl*_.


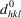
 : equilibrium (unstressed) plane spacing of the [*hkl*] family of planes.


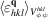
 : average atomic lattice strain along **q**_*hkl*_.

ϕ, ψ: angles describing the orientation of **q**_*hkl*_ in the sample coordinate system **P**.


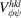
 : total volume of all grains contributing to the *hkl* Bragg peak at the particular orientation defined by the ϕ, ψ angles.


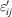
 : local strain tensor components in the laboratory coordinate system **L**.


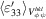
 : components of the average strain tensor for *V*_ϕψ_ in the laboratory coordinate system **L**.


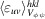
 : components of the average strain tensor for *V*_ϕψ_ in the sample coordinate system **P**.


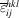
 : reflection average strains for the total reflection volume 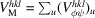 in the sample coordinate system **P**.


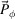
 : direction in the sample coordinate system **P** along which the biaxial stress component 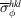 is measured.

DEC: quasi-isotropic diffraction elastic constants 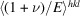 and 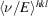 for reflection *hkl*.


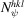
 : number of grains diffracting into a given *hkl* reflection at a given ψ tilt.

α_*t*_: coefficient of linear thermal expansion (CTE) of phase *t*.

ɛ^Th, *t*^: isotropic thermal strain for (cubic) phase *t*.


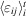
 , 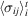 : average strain and stress, respectively, of grain number *i* and phase *t* in the **P** coordinate system.


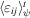
 , 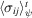 : average strain and stress, respectively, in the **P** coordinate system of the grain set diffracting at a given ψ orientation for phase *t*.

*Z*_I_: Zener index.

## 2. Introduction

The measurement of internal stresses and strains in crystalline materials using diffraction techniques has been widely practised for almost a century (Lester & Aborn, 1925 ▸). There is a broad literature base which supports these measurements, including monographs (Noyan & Cohen, 1987 ▸; Hauk, 1997 ▸; Hutchings *et al.*, 2005 ▸), best-practice manuals (Ahmad *et al.*, 2003 ▸; Fitzpatrick *et al.*, 2005 ▸) and measurement standards (ASTM, 2020 ▸), as well as numerous articles in scientific and trade journals. These measurements have a wide range of technological applications, and are performed in the field and in laboratories to measure applied or residual stresses (Lee *et al.*, 2017 ▸; Ramirez-Rico *et al.*, 2016 ▸).

Since the results of such measurements are utilized in design, quality assurance and failure analysis of critical engineering components, significant effort has gone into their validation and verification. Most of this work was carried out in the last half of the previous century and identified two key issues:

(i) For specimens subjected to directional plastic deformation, such as uniaxial elongation or rolling, the computed stress results did not agree with the results of mechanical relaxation techniques; diffraction-based stress distributions were constant across the sample cross section, which violated the equations of equilibrium. Such non-physical stress distributions were termed ‘fictious’ by some researchers (Cullity, 1964 ▸, 1976 ▸; Abuku & Cullity, 1971 ▸).

(ii) The variation in lattice strains with direction within some samples did not obey the coordinate transformation rules for second-rank symmetric tensors as dictated by elasticity theory; that is, the measured elastic lattice strains did not behave as expected for (second-rank symmetric) tensor elements. This second issue was not limited to specimens subjected to directional plastic flow (Bollenrath *et al.*, 1967 ▸; Taira *et al.*, 1971 ▸; Marion & Cohen, 1974 ▸, 1975 ▸, 1976 ▸).

Different groups attributed these observations to various causes, and over the years many techniques have been proposed to obtain the ‘true’ continuum-mechanics-compatible elastic strain/stress distributions in such samples (Greenough, 1949 ▸; Bollenrath *et al.*, 1967 ▸; Marion & Cohen, 1975 ▸; Peiter & Lode, 1983 ▸; Brakman, 1983 ▸; Peiter, 1986 ▸; Daymond, 2004 ▸; Ortner, 2006 ▸, 2009 ▸, 2011 ▸). These and similar formalisms, however, have not found widespread acceptance, and most have been (effectively) neglected, along with the issues they were designed to address. The great majority of the current analysis algorithms implemented in stress-scanning machines at large facilities such as neutron sources or synchrotrons, or supplied to end-users with laboratory-based or portable diffraction systems, are ‘black boxes’; these do not have explicit provisions to alert end users when the data acquired by such systems do not fit the assumptions inherent in basic theory used in the analysis.

In the current work we investigated how diffraction-based strain/stress results can diverge from those predicted by linear elasticity theory using comprehensive numerical simulations of applied/residual stress distributions. For this purpose, we constructed an ideal polycrystalline model where the shape, orientation and location of all constituent crystallites were specified. This model was populated with randomly placed elastically anisotropic (Cu) or elastically isotropic (W) crystallites for the single-phase simulations, and with Cu and W grains for the two-phase case. Finite-element modelling (FEM) was used to obtain the local stress/strain tensors at all nodes within the models in response to (i) free thermal expansion and (ii) far-field biaxial stress states induced by constrained thermal expansion. These results represent the ‘direct-space’ stress/strain distributions which correspond to stresses determined by mechanical methods (Prime, 2009 ▸; Schajer, 2013 ▸). The local strain tensors were also used to calculate the lattice strains along the various diffraction vectors for the individual grains. These were used to calculate the stress states in various crystal volumes with the traditional diffraction stress analysis formalism. Thus, we could compare the FEM-based stress values with those determined from diffraction analysis.

Our results show that differences in elastic strains/stresses measured with mechanical or diffraction methods originate from the (very) different sampling modes and, consequently, the different averages taken by the respective probes. Results from the two approaches agree if and only if the volumes probed by diffraction are comparable in size or greater than representative volume elements (Bonda & Noyan, 1992 ▸, 1996 ▸) of the particular analysis. If these conditions are not met, measurement of internal elastic strain/stress states using any approach becomes non-trivial and requires information about local grain topology, boundary conditions, gradient parameters *etc.* within the measurement volumes.

## 3. Theory

Most classical residual stress determination formalisms applicable to polycrystalline materials utilize linear elasticity theory, usually with isotropic material constants. These formalisms contain implicit assumptions about the homogeneity of the material and the dimensionality and spatial variation of strain/stress fields in regions of interest. In polycrystalline materials these assumptions are not valid at the local scale but may be ‘statistically’ satisfied for larger domains. These issues are reviewed below. Relevant concepts of linear elasticity analysis, the critical assumptions associated with this analysis and a consistent set of definitions used in this article are summarized in Appendix *A* [App appa].

### 3.1. Stress/strain distributions in heterogeneous materials

Polycrystalline solids belong to the set of heterogeneous materials. In such systems the elastic moduli vary with position in the solid body. Such variation might be systematic, as in fibre or laminate composites, or random, as in non-textured polycrystalline materials. Here we discuss the case of a non-textured single-phase polycrystalline solid subjected to a uniform far-field stress field **σ** ^0^ on its boundary. In such systems the heterogeneous (position-dependent) distribution of elastic moduli within the sample volume results in heterogeneous distributions of stress and strain. Consequently, one must specify the positions or volumes for which stress/strain tensors are reported. In actual direct-space experiments the measured tensors will be averages over the information domain (line, area or volume) sampled by the measurement technique. Diffraction values are volume averages. In what follows such average quantities will be denoted by angle brackets, 〈**σ**〉_*V*_, where any subscript specifies the region within which the particular parameter is averaged. If the average is taken over the entire specimen volume *V* ^0^, the average quantities are indicated by an overline, *e.g.* 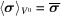 . For diffraction-based averages, any reflection dependencies are specified as superscripts, 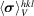 .

**Figure 1:**
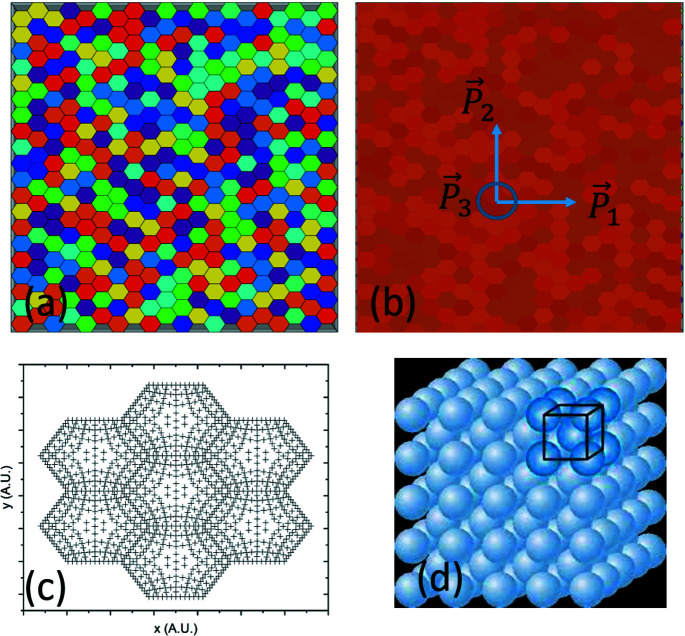
(*a*) An ideal untextured single-phase polycrystalline sample consisting of identical hexagonal prism-shaped anisotropic crystallites; the elastic modulus *C*_*ijkl*_ along any direction varies from grain to grain. (*b*) The equivalent isotropic sample, representing the ideal sample with Young’s modulus ( 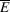 ) and Poisson’s ratio ( 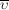 ) determined from averages of the grain compliances over *V* ≥ RVE. (*c*) Finite-element nodes in a cluster of seven grains within the ideal sample. Here each (node) point within a particular grain is a material point and the material is assumed to be continuous between these nodes. (*d*) In a real crystalline material many unit cells are needed to constitute such a material point. The same sample coordinate system **P** is used for panels (*a*), (*b*) and (*c*).

Consider an ideal single-phase polycrystalline body where all crystallites are of the same shape and size. A cross section of such a sample, based on hexagonal prism-shaped crystallites, is shown in Fig. 1 ▸(*a*). We assume that: (i) the dimensions of the body are many orders of magnitude larger than the dimensions of its constituent crystallites, and all crystallites have dimensions which are several orders of magnitude larger than the unit-cell parameters of the material; (ii) all unit cells in all crystallites are ‘perfect’ such that, using symmetry considerations, any given crystallite can be replaced by a homogeneous continuum with identical stiffness/compliance tensors at all points within it; (iii) each crystallite is solidly bonded to all of its neighbours across mutually shared facets with no gaps; (iv) the boundaries between crystallites (grain boundaries) are infinitesimally thin perfect geometric planes; (v) any internal loads or displacements are completely transferred across these boundaries; and (vi) all crystallites are randomly oriented in space with respect to a single Cartesian sample coordinate system **P** (with 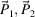 in the sample surface) and there is no correlation in the lattice orientations of neighbouring crystallites. For simplicity, we further assume that the unit cell of the material has cubic symmetry.

If this material is subjected to a uniformly distributed external plane stress field **σ** ^0^, the local stress and strain matrices at any point *Q* are linked by the local elastic moduli **C**(*Q*) and **S**(*Q*),

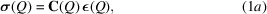




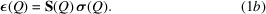

Here **C** and **S** are symmetric 6 × 6 matrices (replacing fourth-rank tensors) which are defined for the particular crystallite (containing point *Q*) in the *P_i_* coordinate system (Nye, 1985 ▸). Since **C** and **S** change from grain to grain, local terms are heterogeneously distributed in the material volume (between and within grains) to satisfy the compatibility and equilibrium conditions (Appendix *A* [App appa]). In general, these terms are not equal to their far-field values. However, it is possible to homogenize these terms by considering their average values over length, area and volume elements.

In the volume *V* ^0^ of the ideal specimen, any vector 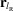 of sufficient length, ||**r**|| = *l*_R_ or greater, will sample (traverse) a sufficient number *N*_R_ or more of (random) crystal orientations along its length, such that the average elastic moduli along 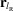 will tend to the volume-averaged elastic moduli of the entire specimen, 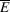 and 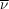 . We define *l*_R_ as the ‘representative line element’ (RLE). The stress–strain response along any vector **r** with ||**r**|| ≥ *l*_R_ can be represented by a material point (defined formally in Appendix *A* [App appa]) in an equivalent isotropic material [Fig. 1 ▸(*b*)] with elastic moduli 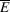 and 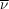 . In two and three dimensions, the ‘representative area element’ (RAE) and ‘representative volume element’ (RVE) are similarly defined (Bonda & Noyan, 1992 ▸; Salahouelhadj & Haddadi, 2010 ▸; Harris & Chiu, 2015*a* ▸,*b* ▸; Marino *et al.*, 2019 ▸).

Following Hill (Bishop & Hill, 1951 ▸; Hill, 1952 ▸), the average elastic moduli can be defined by considering the energy density in the equivalent elastic material with stress and strain fields **σ** ^0^ and **ε** ^0^,

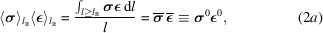




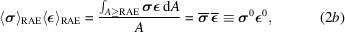




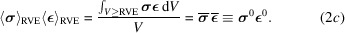




The product **σ** ^0^ **ε** ^0^ is twice the actual energy density of the sample defined in terms of measured macroscopic quantities. Hill used equation (2*c* [Disp-formula fd2c]) to show that the average Young modulus 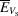 of an ideal single-phase polycrystalline sample falls between the Voigt and Reuss limits (constant strain and constant stress in all grains, respectively) and can be approximated by the arithmetic mean of the elastic moduli for these limits, provided that (i) the grain orientations in the material are random and (ii) the sample is large enough with respect to the grain size that all possible orientations are adequately sampled. This ‘arithmetic mean’ approximation had been experimentally demonstrated a decade earlier by Neerfeld (1942 ▸).

In summary, as long as the strain response of an ideal single-phase polycrystalline sample is measured in direct-space domains equal to or larger than the representative domains for the particular dimension, by a linear strain gauge along any RLE, by an area strain gauge on any REA, or by a volumetric strain measurement technique over any RVE, the measured response will be ‘isotropic’ and the applied far-field stress can be linked to these average strain tensors through Hooke’s law. In this context, material domains larger than the representative elements are termed ‘quasi-isotropic’ (or ‘statistically isotropic’). If the material is textured, similar representative elements can be specified wherein all texture components of an infinitely large bulk sample are adequately represented. For such cases material domains larger than textured representative elements can be termed ‘quasi-anisotropic’ (or ‘statistically anisotropic’). Hill designates such material volumes as ‘macroscopically homogeneous’ (Hill, 1952 ▸); these can be represented by equivalent homogeneous materials or material points. We note that the procedure described above contains an implicit prior homogenization step: the geometric points (nodes in a finite-element mesh) in each grain under analysis [Fig. 1 ▸(*c*)] correspond to volumes of material containing, at a minimum, representative numbers of unit cells [Fig. 1 ▸(*d*)], such that each point possesses the macroscopic physical properties of the particular grain and can be considered a ‘material point’.

### 3.2. Scale-dependent stress/strain definitions in quasi-homogeneous materials

Using the discussion on representative domain elements one can define three direct-space domain scales for linear elasticity analysis in the ideal polycrystalline sample shown in Fig. 1 ▸(*a*):

*Scale 1.* For the set of material points {*Q*} within any crystallite in the sample volume, the anisotropic Hooke law links the local stresses and strains **σ** and **ε**. To a first approximation these terms can be expressed as perturbations around the average stress/strain values, respectively, referred to the entire sample volume:

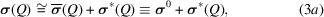




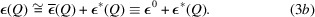

Here, σ* and ε* are *position-dependent* (local) interaction stress/strain matrices arising to satisfy equations of force equilibrium and displacement compatibility during the collective deformation/distortion of the (bonded) aggregate of anisotropic crystallites. These terms were first described by Heyn in 1914 (Heyn, 1914 ▸; Masing, 1923 ▸). Following Garrod & Hawkes (1963 ▸) and Krier *et al.* (1991 ▸), we term σ* and ε* as Heyn stresses and strains, respectively. If the local (total) strain **ε** at a material point could be measured, the local stress **σ** could be computed from the (general) Hooke law using the appropriate single-crystal stiffness matrix **C** for the particular grain. Then, if the far-field stress and strain terms **σ** ^0^ and **ε** ^0^ are known, the Heyn interaction terms σ* and ε* at a particular point *Q* can be computed.

*Scale 2.* If the measurement/analysis volume *V*_M_ is larger than the direct-space representative volume element RVE_DS_ then the interaction stress/strain matrices integrate to zero,

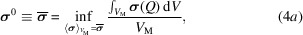




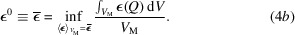




Equations (1*a* [Disp-formula fd1a]), (1*b* [Disp-formula fd1b]), (4*a* [Disp-formula fd4a]) and (4*b* [Disp-formula fd4b]) indicate that the elastic moduli linking 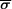 and 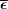 in RVE_DS_ must be isotropic and equal to the bulk elastic moduli 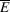 and 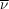 . Thus, the average strain measured in such cases, 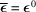 , could be directly substituted in the isotropic Hooke law with 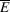 and 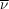 to obtain the average stress 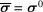 . In such a measurement the interaction strain/stress terms are not available. If, after such an experiment, only 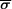 and 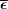 are reported, the polycrystalline material volume is implicitly represented as being ‘macroscopically isotropic’ and can be represented by an equivalent homogeneous material with elastic moduli 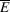 and 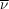 for further design, failure analysis or ‘lifing’ considerations.

**Figure 2:**
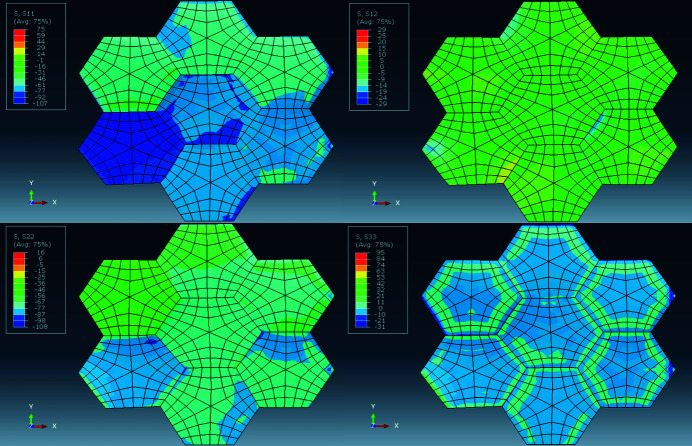
Contour maps of nodal stresses (clockwise from top left) σ_11_, σ_12_, σ_33_ and σ_22_ with node position for a meso-scale volume containing seven anisotropic Cu grains. These were obtained from FEM analysis (Section 4) when the polycrystalline mesh shown in Fig. 1 ▸(*a*) was subjected to uniform (isotropic) biaxial far-field stresses. Averaging these local stress tensors over individual grains, or over this meso-domain, did not eliminate Heyn stresses; the stress distributions in element, grain and meso-domain volumes were not isotropic in the slab plane.

*Scale 3.* Meso-scale (MS) domains smaller than RVE_DS_ contain statistically unrepresentative numbers of grains and other microstructural features. Consequently, the volume averages of the interaction stress and strain fields may not vanish, and the domain-averaged stress and strain values 〈**σ**〉_MS_ and 〈**ε**〉_MS_ need not be equal to the far-field values **σ** ^0^ and **ε** ^0^ (Fig. 2 ▸). It follows that the average elastic moduli 〈**C**〉_MS_ and 〈**S**〉_MS_ of such domains will contain domain-specific configurational terms. These cannot be computed using only the moduli of the constituent grains; all grain orientations, geometries, topologies and interface properties must also be specified. Also, one cannot obtain true elastic constants for meso-domains by linking applied (far-field) stresses **σ** ^0^ to measured local average strains 〈**ε**〉_MS_: since the average interaction strains are finite, the computed moduli will contain configurational components and thus will deviate from ‘true’ elastic moduli. Consequently, computation of the average stress 〈**σ**〉_MS_ in an arbitrary meso-domain from measured average strains is non-trivial.

The stresses and strains for all three domain scales discussed above are symmetric second-rank tensor quantities and can be transformed into other coordinate systems. For the first and third cases these transformations are strictly defined only at the particular point *Q* or for the centre of mass of the particular meso-domain, respectively. These transformed tensor quantities cannot be generalized to any other point, any other meso-domain or the entire sample without further analysis. In addition, the dimensionalities of stress/strain tensors for Scales 1 and 3 are not necessarily identical to the far-field stress tensor (in Fig. 2 ▸ the far-field load is isotropic plane stress). For material volumes larger than RVE_DS_ Scale 2 applies; the average stress/strain tensors for all RVE_DS_ are identical to those of the equivalent representative material and have the same form as any far-field stress/strain tensors.

### 3.3. Saint-Venant effects

The discussion so far has considered stress/strain distributions in the central regions (distant from boundaries) of (large) solid parts subjected to uniformly applied far-field loads. For samples with point loads and tractions, internal or surface geometric discontinuities *etc.*, local strain/stress gradients can form (Appendix *B* [App appb]). The Saint-Venant principle asserts that these gradients vanish at sufficiently large distances (≥ Saint-Venant length) from loading points and other singularities (Toupin, 1965 ▸).

For polycrystalline materials, the strains/stresses caused by such end effects will be superimposed on the Heyn strains/stresses arising from the heterogeneous distribution of elastic moduli and plastic flow. Thus, the local stress and strain values at a given point *Q* can be written as

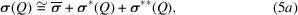




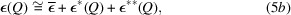

where σ**(*Q*) and ε**(*Q*) are the local Saint-Venant strains and stresses, respectively.

The presence of end effects has several ramifications. First, for the volumes in such regions where there are stress/strain singularities (point loads, sharp crack tips *etc.*), higher gradients of the displacement field must be included in the expression for the strain energy function. Thus, formally, basic linear elasticity theory is not applicable at such points. Second, in Saint-Venant regions a single stress/strain measurement is not sufficient to infer the far-field load distribution (Appendix *B* [App appb], Fig. 16). Third, experimentally defining an RVE_DS_ in such regions is complicated. For example, in polycrystalline mater­ials it is possible to use texture measurements or electron backscatter diffraction orientation mapping to define domains within Saint-Venant regions in which the elastic moduli are statistically homogeneous, equal to those of any RVE_DS_ in central regions and identical to the elastic moduli of the corresponding equivalent homogeneous mater­ial. However, because of stress/strain concentration issues, linking the strain tensors for such domains to applied (far-field) stresses is non-trivial; integration over the entire Saint-Venant volume is needed to average out the effects of stress concentration.

Finally, specification of a local measurement volume in Saint-Venant regions requires care. First, it may not always be possible to infer the stress state of such regions by inspection. Second, in regions of interest where steep strain gradients exist, a small measurement volume might not contain enough grains to constitute an RVE, introducing meso-domain issues. Specification of a larger measurement volume to overcome this issue would average over a large portion of the steep strain gradient. In both cases such a measurement could yield average strain/stress tensors which are not representative of either the local or the far-field strain/stress tensors. In the case of diffraction measurements, the relevant sampling issues are exacerbated by the physics of the diffraction process. These are discussed next.

### 3.4. Diffraction-based strain measurements

We limit our discussion to monochromatic diffraction measurements performed on polycrystalline materials. The (much) abbreviated treatment in this section is based on the monograph by Noyan & Cohen (1987 ▸). It is extended to state explicitly the major assumptions in this analysis, in particular those which are relevant to applications of this formalism to polycrystalline materials.

**Figure 3:**
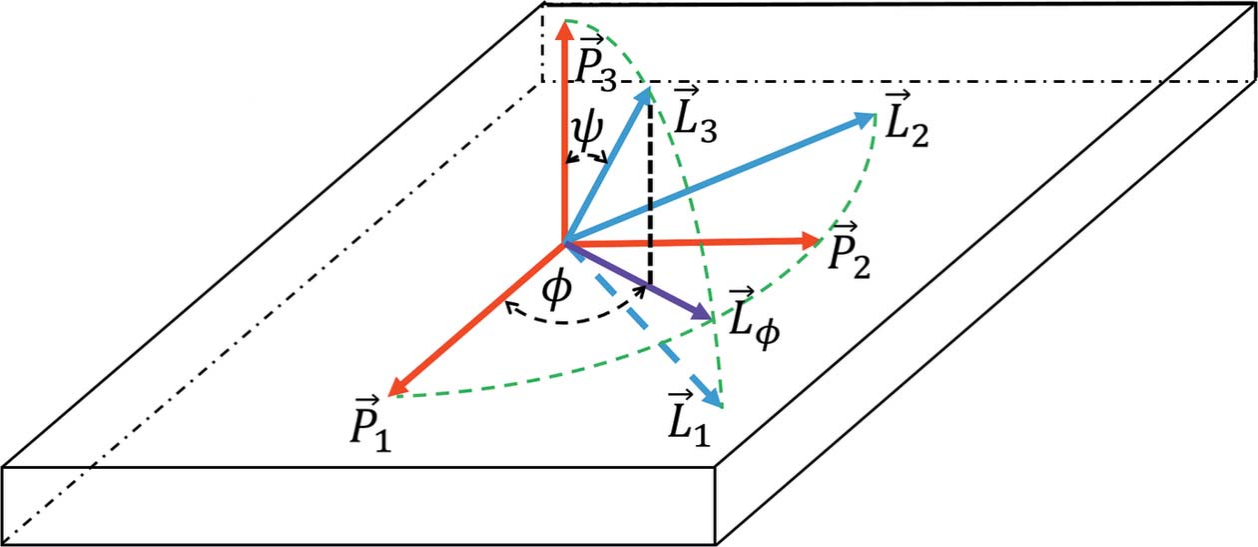
Definition of the angle ϕ and orientation of the (orthogonal) laboratory coordinate system **L** with respect to the sample coordinate system **P**. Vectors 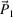 , 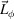 , 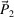 and 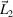 are in the plane of the sample surface. Vectors 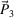 , 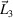 , 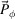 and 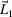 are coplanar.

It is assumed that appropriate collimators are used to limit the acquired diffraction signal to a particular region (volume) of interest (ROI) and that the peak belonging to a single reflection *hkl* originating from the ensemble of grains within the ROI is analysed. In this ‘single-peak’ analysis, the atomic plane spacing 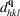 of a particular family of planes {*hkl*} along the specific scattering vector **q**_*hkl*_ is obtained from Bragg’s law,

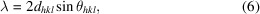

where θ_*hkl*_ is the Bragg angle for the particular peak. The scattering vector **q** is defined as **q**_*hkl*_ = **k**_d_ − **k**_0_, where **k**_d_ and **k**_0_ are the wavevectors of the diffracted and incident rays, respectively. The vector **q**_*hkl*_ is perpendicular to the diffracting planes, and **q**_*hkl*_, **k**_d_ and **k**_0_ are coplanar. In the sample coordinate system **P**, the scattering vector **q**_*hkl*_ is described by two measurement angles, ϕ and ψ (Fig. 3 ▸).

The lattice strains are then computed from 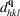 ,

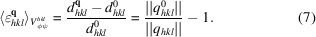

Here 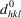 is the equilibrium (unstressed) plane spacing and the computed (lattice) strain is a diffraction average over the total volume 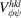 of all grains contributing to the *hkl* Bragg peak at the particular orientation defined by the ϕ, ψ angles. We term 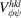 a ‘ψ volume’, where for brevity the ϕ dependency is not explicitly stated (but is assumed).

Defining the (orthogonal) laboratory coordinate system **L** such that 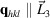 , with 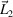 in the plane defined by 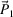 and 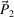 , one can relate the strain tensors in the **L** and **P** coordinate systems using the second-rank tensor transformation rule (Appendix *A* [App appa]),

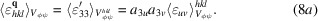

Here, the primed average strain 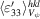 is defined along 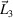 and (unprimed) strains 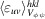 are defined in the sample coordinate system **P** averaged over the grain volumes contributing to the Bragg peak at sample orientation ϕ, ψ. Substituting for the direction cosines *a*_3*u*_ and *a*_3*v*_,

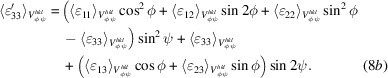




Equation (8*b* [Disp-formula fd8b]) has six unknown variables 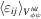 and one measured parameter 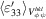 . The classical analysis uses six or more independent 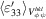 values, measured at various independent orientations ϕ, ψ, in a simple least-squares regression analysis to obtain the strains 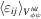 in the sample coordinate system. This approach involves the specification of six independent laboratory coordinate systems **L**_*i*_ (*i* = 1–6), each with its unique measurement angles ϕ_*i*_, ψ_*i*_, and posits that (i) all coordinate systems **L**_*i*_ and **P** share the same origin and (ii) all 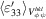 are elements of the same (symmetric) second-rank tensor. [Equation (8*a* [Disp-formula fd8a]) implicitly defines diffraction-based strains as components of a second-rank tensor, an array of numbers that transform according to the specified transformation rules under a change of coordinates (Nye, 1985 ▸).]

These assumptions are trivially satisfied for a homogeneous material. For polycrystalline materials, however, the grain populations belonging to each ψ volume 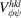 are mostly independent; different grains satisfy the Bragg condition at different angle pairs ϕ_*i*_, ψ_*i*_ (Noyan & Nguyen, 1987 ▸, 1988 ▸; Chidambarrao *et al.*, 1997 ▸). Consequently, equation (8*b* [Disp-formula fd8b]) links the average strain tensor associated with each **L**_*i*_ to the average strain tensor associated with a unique laboratory coordinate system **P**_*i*_ defined at the centre of mass of the grain population diffracting at orientation angles ϕ_*u*_, ψ_*u*_. Simultaneous solution of the set of these equations assumes that the average strain tensors for the grain sets in the diffraction condition are identical for all **P**_*u*_; 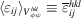 for all elements of 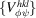 ,

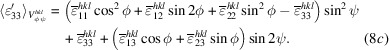




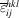
 are the reflection-average strains defined for the total reflection measurement volume 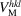 from which 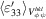 data are obtained, with 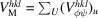 ; *U* is the number of sampled orientations, *u* ∈ (1, *U*).

Equation (8*c* [Disp-formula fd8c]) shows that the variation in 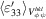 with 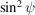 must be linear when both shear strains 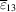 and 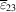 are zero [for brevity, we neglect any curvature due to stress gradients 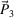 normal to the surface (Noyan, 1983*a* ▸); this simplification does not materially change any conclusions]. Such plots can be analysed for 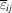 using simple linear regression of 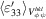 versus 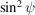 data. If either (or both) of the shear strains 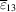 and 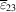 are finite, the 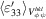 versus 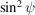 plot will exhibit ‘ψ splitting’ for  ∓ ψ values due to the 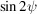 term. The analysis in this case is also based on linear regression, with additional computation of some intermediate terms (Noyan & Cohen, 1987 ▸). The average stresses 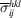 in the reflection measurement volume 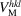 can then be computed from Hooke’s law with appropriate elastic constants for the diffracting ensemble.

If the polycrystalline material within the reflection measurement volume 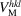 is statistically isotropic, the average strain terms in equation (8*c* [Disp-formula fd8c]) can be expressed in terms of average stresses in the sample coordinate system **P** using the isotropic form of Hooke’s law,

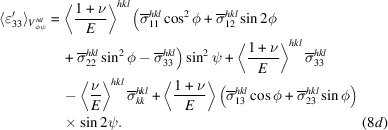

The reflection-average stresses 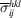 are assumed to be constant in all ψ volumes, 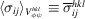 .

The terms 〈(1 + ν)/*E*〉^*hkl*^ and 〈ν/*E*〉^*hkl*^ are diffraction elastic constants (DECs) and depend on the Miller indices *hkl* of the reflection used for obtaining 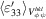 . These can be computed from single-crystal moduli using various approximations (Noyan & Cohen, 1987 ▸). The reflection dependency arises from the Bragg selectivity of the diffraction process; for each diffracting grain within 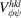 , one member of the *hkl* family of directions must be coincident with the laboratory axis 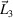 . Thus, the set of diffracting grains for each ψ volume defines a virtual fibre-textured ensemble with fibre axis [*hkl*]. For random polycrystalline materials the (computed) DECs do not depend on the measurement angles ϕ, ψ since the (independent) fibre-textured ensembles selected at each ψ tilt are assumed to be identical.

In most practical measurements utilizing X-rays with energies below 10 keV, the stress terms in the direction of the sample normal 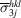 (*j* = 1–3) in equation (8*d* [Disp-formula fd8d]) are assumed to be zero due to the shallow penetration depth of the radiation. In this case equation (8*d* [Disp-formula fd8d]) is further simplified,

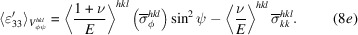

Here 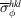 is the in-plane stress along the surface direction 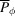 (Fig. 3 ▸).

Equation (8*e* [Disp-formula fd8e]) also predicts linear 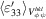 versus 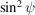 . From the slope of the fitted line one can directly compute the in-plane stress 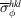 using the appropriate DEC. This is the classic ‘ 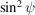 ’ method.


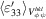
 versus 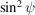 plots which do not exhibit regular behaviour (linear or ψ split), such as those exhibiting statistically significant oscillations, indicate that the average strain states in all diffracting volumes sampled during the experiment 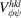 are not identical, *i.e.* 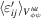 are heterogeneously distributed in the total sampled reflection volume *V* ^*hkl*^,

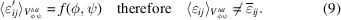

In such cases the average strains 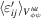 predicted by equation (8*c* [Disp-formula fd8c]) and its extensions deviate significantly from measured 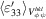 versus 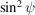 data. Such deviations might occur if, for example, 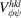 do not constitute representative volume elements.

### 3.5. Defining representative volume elements for diffraction measurements

There are numerous experimental and numerical studies of representative volume elements RVE_DS_ in direct space (Bonda & Noyan, 1992 ▸, 1996 ▸; Harris & Chiu, 2015*a* ▸,*b* ▸; Vel *et al.*, 2016 ▸; Marino *et al.*, 2019 ▸). In contrast, there has been very little discussion of the conditions under which grain ensembles sampled in diffraction strain/stress measurements constitute (diffraction) representative volume elements 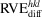 .

Consider an ideal untextured single-phase polycrystalline material with anisotropic grains subjected to uniform far-field plane stresses at its boundary [Fig. 1 ▸(*a*)]. Any direct-space measurement volume 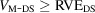 can be represented by an equivalent isotropic material volume or material point with elastic moduli 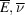 linking the far-field strains and stresses **ε** ^0^ and **σ** ^0^ in 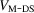 . When diffraction is used to illuminate 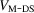 to obtain a set of average strain data 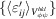 along independent 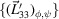 , these data originate from a set of independent ψ volumes 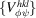 . These, and the total reflection volume 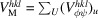 , are (proper) subsets^1^ of 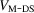 , 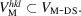 If the (measured) variation in 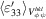 with 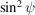 is linear and 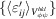 are used, with the appropriate form of equations (8*a* [Disp-formula fd8a])–(8*e* [Disp-formula fd8e]), to compute the average stress tensor 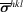 in the sample coordinate system **P**, it is assumed that 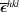 , 〈(1 + ν)/*E*〉^*hkl*^, 〈ν/*E*〉^*hkl*^ and 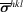 will be identical for all 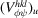 (*u* → ∞) which could be measured within the direct-space volume 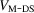 for a given reflection *hkl*. Thus, the total reflection volume 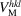 is assumed to be a ‘virtual’ homogeneous material with ideal fibre texture (with texture axis [*hkl*]) subjected to uniform far-field stresses on its boundary. This is the fundamental assumption of diffraction strain/stress analysis using a single reflection. Since this assumption is only asserted for the particular reflection, it is not intimated that 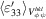 versus 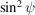 data from other reflections must also exhibit regular behaviour or that all reflection volumes have identical stress/strain states.

Consider now the case where two (or more) independent reflections are used to obtain independent linear 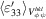 versus 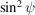 plots within the direct-space volume 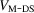 . Thus, each sampled reflection volume 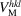 contains its own homogeneous strain/stress distributions. If statistically representative grain populations are sampled for each reflection, such that any Heyn stresses are averaged out, the average stresses computed for each reflection, 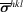 and 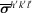 , would be equal to the far-field stress **σ** ^0^ acting on the boundaries of the real-space volume 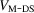 . However, for independent reflections, the diffraction elastic constants 〈(1 + ν)/*E*〉^*hkl*^ and 〈ν/*E*〉^*hkl*^ are not identical or equal to the bulk values. Thus, if 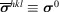 , then 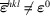 . This is physically possible, since 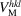 are distributed interpenetrating volumes in direct space; fibre-textured volumes 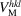 corresponding to each reflection, each with a different average strain 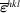 , cannot be continuous direct-space volumes within 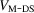 due to Saint-Venant compatibility conditions.

On the basis of this discussion, a diffraction representative volume 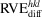 for an untextured single-phase material must satisfy the following conditions:

(i) The variation in 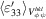 versus 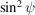 measured from grains within 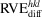 for the particular reflection must be linear (regular) within experimental error.

(ii) 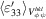 values must be stable with grain population: for a given illuminated volume, the addition of more grains for a particular *L*_ϕ,ψ_ orientation, or sampling additional grains at new independent orientations, should not change the results of the regression analysis outside error bounds.

(iii) The average stress tensor 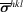 computed from the diffraction data using Neerfeld–Hill or Kröner DECs must be equal to the far-field stress tensor **σ** ^0^ acting on the boundaries of the smallest direct-space volume 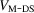 containing the set of diffracting grains described by Conditions (i) and (ii). We note that specifying 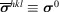 for all *hkl* does not indicate that the material is at the Reuss limit; each point within 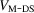 has heterogeneous stress/strain distributions which are averaged to zero for 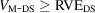 .

For an ideal non-textured single-phase polycrystal with equiaxial grains and a unimodal size distribution, all reflections would be expected to have similar 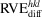 . For a real sample, however, one needs to test whether, for each reflection, the measured data originate from an 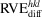 . For cases where the far-field stress is known, all three conditions can be tested experimentally. For samples with significant intergranular residual stress distributions, the first two conditions might be relatively easy to test, but verifying the third condition, 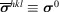 , might be problematic. If multiple reflections in a single-phase material yield the same stress value, 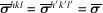 , it is more likely that 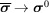 ; this is a necessary but insufficient condition. However, this test is inapplicable to multi-phase materials. Consider a two-phase random polycrystalline aggregate loaded into the plastic flow regime, where one phase is much weaker. While a strain gauge mounted on such a sample will average over strains in both phases, diffraction can only interrogate one phase at a time: Heyn stresses arising in response to the systematic partitioning of plastic deformation cannot be averaged out within a single phase. Thus, the stress/strain tensors measured by diffraction from each phase will not be equal to the (direct-space) far-field stresses acting on the specimen boundary. Similar issues can also arise in textured single-phase materials, since slip and anisotropic elastic moduli depend on crystallographic orientation; texture components with different mechanical responses can act as virtual phases.

The discussion presented above shows that specification of representative volume elements in diffraction measurements does not depend only on ‘elastic anisotropy’. The issue can be quite challenging if plastic deformation is systematically partitioned between crystal groups of different orientations and/or between different phases. To investigate these issues, we used finite-element modelling.

## 4. Finite-element modelling

The basic modelling approach is based on our previous work (Noyan & Nguyen, 1987 ▸, 1988 ▸; Chidambarrao *et al.*, 1997 ▸), where partitioning of elastic strains in finite-element models of fully anisotropic crystallites was described. We prefer this approach rather than that of spherical anisotropic grains embedded in an isotropic continuum (Ruppersberg *et al.*, 1989 ▸; Krier *et al.*, 1991 ▸), since the FEM code computes position-dependent Heyn stresses and strains, as well as plastic strain distributions, if any, resulting from the ‘compatible’ deformation of the total aggregate, and enforces compliance with both the equations of (static) equilibrium and the compatibility conditions. In the current study we used the *ABAQUS/CAE* suite (Dassault Systèmes) to model un­textured polycrystalline single-phase Cu and W, and two-phase Cu–W polycrystalline slabs (W and Cu are insoluble in each other; Vijayakumar *et al.*, 1988 ▸), under free and constrained thermal expansion. Relevant material properties are listed in Table 1 ▸. As shown by its Zener index *Z*_I_, Cu is highly anisotropic. The elastic response of stand-alone W grains is essentially isotropic. Thus, this selection covers most of the elastic anisotropy range for (cubic) metals.

The single-phase slab models consisted of 400 hexagonal prism-shaped anisotropic grains arranged in a 20 × 20 × 1 matrix [Fig. 1 ▸(*a*)]. For the two-phase models the mesh contained 676 grains distributed (almost) equally between the phases. In both cases these (anisotropic) hexagonal grains were complemented by partial grains at the edges [shaded grey in Fig. 1 ▸(*a*)] to obtain a smooth boundary. The partial grains had isotropic elastic moduli equal to the bulk moduli of the modelled material. We used C3D20 elements (20-node linear brick with 27 integration points) for discretization. Each grain was meshed with 540 elements: 48 elements (24 × 2) for the top and bottom surfaces, 300 elements (50 × 6) for side facets, and 192 elements fully internal within each grain. Each grain was ten elements thick. The grain thickness (ten FEM units) was equal to the maximum diameter of the (hexagonal) base. All grain surfaces were connected to each other with tie constraints. To prevent global displacement of the model, explicit equation constraints were used. Thus, the model describes a static single-layer polycrystalline slab with identical quasi-equiaxial grains rigidly bonded across the congruent grain surfaces.

Homogeneous temperature fields were applied over the entire model to induce thermal strains. Since the coefficient of thermal expansion is isotropic in cubic materials, free expansion of these models did not induce interaction stresses between grains. To induce elastic strain fields we used symmetric distributed constraints to fix the boundaries in the plane of the slab. Thus, the mesh was placed under an isotropic compressive far-field plane stress state upon heating. For all models the *ABAQUS* rate-independent isotropic plasticity model was used, with uniaxial yield points of 245 and 750 MPa for for Cu and W phases, respectively. Each grain was assigned its own (maximum) Schmid factor computed from its crystallographic orientation in the sample coordinate system. Any grain within which the total stresses (far-field plus Heyn) reached the Mises yield surface deformed plastically. We did not implement strain hardening since our model was limited to small (≲0.3%) plastic strains.

To specify the crystallographic directions coincident with the axes of the sample coordinate system for crystallites diffracting into a given *hkl* reflection for various ψ angles we used the formalism described by Song & Noyan (1996 ▸). Since the diffraction vector **q** = *h* **x** + *k* **y** + *l* **z** is defined in the sample coordinate system, where **x**, **y**, **z** are the unit vectors along 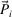 , the crystallographic vector *a*_1_ **x** + *b*_1_ **y** + *c*_1_ **z** along 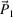 for any ψ (for ϕ = 0) is given by

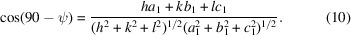

This equation can be solved numerically for the indices *a*_1_, *b*_1_ and *c*_1_ to obtain various vectors along 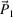 . The crystallographic vectors along 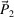 and 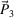 can then be defined by taking the cross products of **q** and 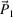 and, once this is done, of 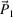 and 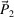 , respectively.

In the present study, these calculations were carried out for the 222, 200 and 420 reflections for eight ψ angles between 0 and 71.57°. We selected grains to yield reasonably uniform in-plane orientations around the diffraction vector for each ψ angle, thus approximating a virtual fibre texture ensemble for each ψ angle. In the model we define a ‘ψ ensemble’, with volume 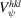 and population 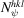 , as the set of grains diffracting into a given *hkl* reflection at a given ψ tilt. Reflection ensembles, with populations *N* ^*hkl*^, refer to the total number of grains scattering at all modelled ψ tilts for a given reflection. Since the volume of each grain is the same, the volume and number fractions 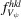 and *f* ^*hkl*^ are identical for ψ volume and reflection ensembles, respectively. Actual grain distribution parameters for all three reflections are shown in Table 2 ▸.

**Figure 4:**
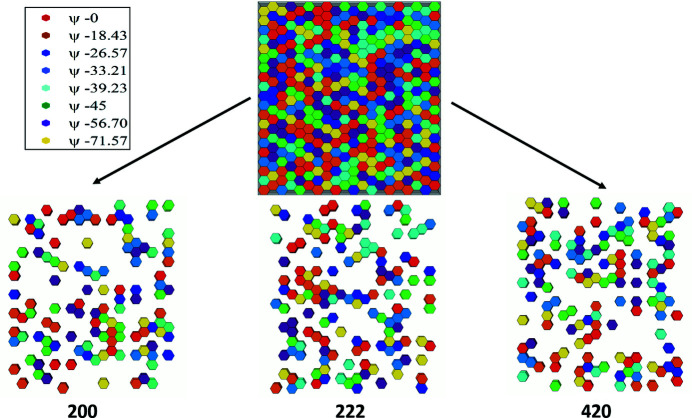
Spatial distribution of the grains scattering into different *hkl* reflections in the mesh. These ‘reflection volumes’ 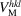 are discontinuous in direct space. Colours indicate the ψ angles at which the diffraction condition would be satisfied for the particular reflection. For each reflection, grains of a given colour constitute a ‘ψ ensemble’ with volume 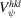 and population 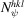 .

These grains were placed randomly in the mesh. The direct-space locations of grain populations for these three reflections are shown in Fig. 4 ▸. The strain tensors measured by diffraction for these populations would be associated with the coordinates of the centre of gravity of the relevant ensembles (ψ volumes or reflection volumes, respectively).

We note that, while in our previous work some grains within the FEM meshes were non-diffracting (Noyan & Nguyen, 1987 ▸, 1988 ▸; Song & Noyan, 1996 ▸; Chidambarrao *et al.*, 1997 ▸), all complete grains in the current models are accessible by diffraction. This approach was chosen so that diffraction stress/strain averages over the entire model could be computed and compared with direct-space values. This approach also enabled us to check whether the average stresses determined from diffraction analysis satisfied the average equilibrium conditions reported previously (Noyan, 1983*b* ▸).

## 5. FEM results

### 5.1. Single-phase models

#### 5.1.1. Unconstrained heating

We first simulated unconstrained expansion of the single-phase models consisting of W or Cu grains subjected to uniform temperature increases. These models served as initial tests of our model.

*Direct-space analysis.* Since the slabs were free to expand along the sample axes, 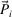 (*i* = 1–3), at all points and the CTEs of both W and Cu are isotropic, the distribution of thermal strains 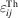 in the mesh must also be isotropic, and no interaction strains, on any scale, should occur. Consequently, thermal strains at any point 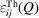 within such models should be independent of position and equal to the strain within an equivalent isotropic sample, 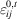 = α_*t*_Δ*T* (*t* = Cu, W). The average strain tensor 〈ɛ_*ij*_〉_*V*_ computed for any node or volume element within a model, or over the entire model, should also be equal to 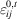 ,

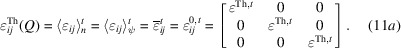

Here, 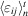 and 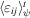 denote the average strains, in the **P** coordinate system, for the *n*th grain and for the grain set diffracting at a given ψ value of phase *t*, respectively.

Since all terms of the strain tensor shown in equation (11*a* [Disp-formula fd11a]) are thermal eigenstrains (Mura, 2013 ▸), the local and average stress components at all scales discussed above must be zero,

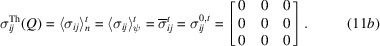




In both cases, finite-element simulations yielded the expected local and average strain and stress values. These average values had zero dispersion, indicating that all nodes and all grains had the same thermal strains and were stress free.

**Figure 5:**
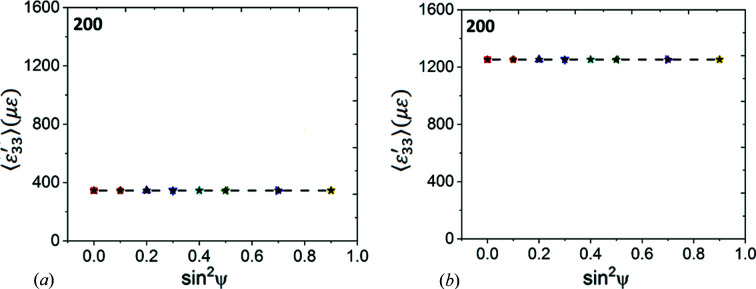
Computed 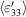 versus 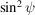 plots for all grains contributing to the 200 reflections of (*a*) W and (*b*) Cu models heated without boundary constraint for Δ*T* = 70°C. Strain values for all grains diffracting at all ψ angles are identical and fall on top of each other. Identical plots were obtained for all reflections of each material.

*Diffraction analysis.* We computed the 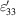 versus 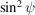 plots for all modelled reflections from the average strains in the diffracting crystallites for the particular ψ volumes. These were all straight lines with zero slopes (Fig. 5 ▸), indicating that diffraction stress analysis would have yielded zero stress [equation (8*e* [Disp-formula fd8e])] irrespective of the diffraction elastic constants used in the analysis (Tables 3 ▸ and 4 ▸). In summary, our unconstrained thermal expansion models of Cu and W samples yielded the predicted stress/strain states, indicating that there were no basic problems with the FEM mesh. In the second set of simulations, stresses were induced in the slabs through constrained thermal expansion.

#### 5.1.2. In-plane constrained heating

In these models we specified homogeneous displacement constraints at the model boundaries in the slab plane and applied uniform temperature fields. Thermal expansion in the grains could only occur normal to the slab surface 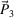 ; the in-plane displacements were kept at zero by reaction forces distributed uniformly over the model boundaries. We first report results from the W model; in this case the expected strain/stress states could be analytically predicted.

(*a*) *Single-phase W slabs.* Since all W grains are isotropic in both elastic and thermal response (Nye, 1985 ▸), the elastic moduli at all points within the model will be independent of orientation and all points will undergo identical displacements in response to heating. Thus, the local strain tensor at any point *Q* within the W model is expected to be homogeneous with the form

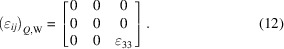

The total lattice strain (ɛ_*ij*_)_*Q*,W_ along the sample normal 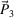 at any node *Q* in the W mesh is the sum of two components: an elastic strain 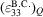 caused by the boundary constraint in the plane of the slab, plus the thermal strain ɛ^Th,W^ due to the temperature increase. From basic elasticity analysis we obtain

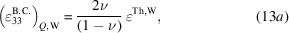




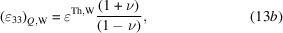




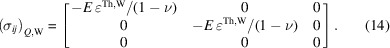




*Direct-space analysis.* The stress and strain values in the sample coordinate system, computed using these equations for our W model, are listed in Table 5 ▸ for a temperature increase of 70°C. The corresponding results from the FEM analysis are also included. We observed excellent agreement between the values obtained from the FEM simulation and the analytical computations. As expected, the FEM simulation yielded isotropic stress and strain distributions within the model volume; all average stresses and strains were identical to their local (node) values, independent of the type of averaging and the size and location of the averaging volume, and all exhibited zero dispersion. Since the induced stresses are much lower than the uniaxial yield point of W, there was no plastic flow in this model.

**Figure 6:**
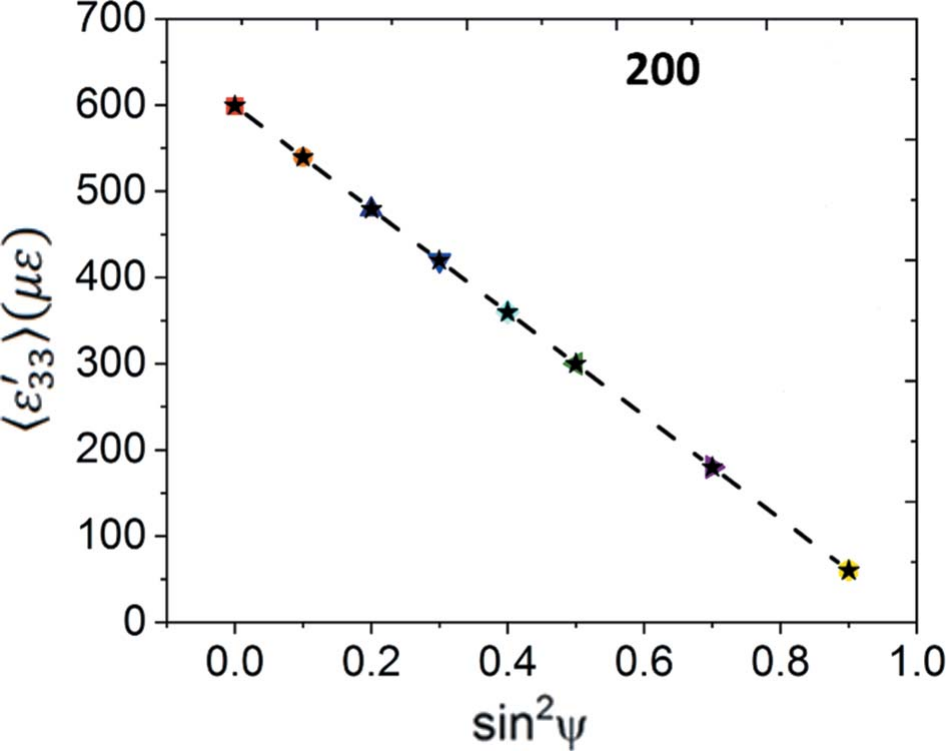
Computed 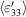 versus 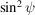 plot for all grains contributing to the 200 reflections of the edge-constrained W model subjected to a temperature increase of 70°C. For each ψ angle, all grains in the diffraction condition had identical 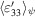 . The exact same plot was obtained for all W reflections.

*Diffraction analysis.* The stresses in the sample coordinate system were also calculated by simulating and analysing 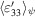 versus 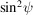 graphs (Fig. 6 ▸) for all reflections. In all cases these plots were linear with identical slopes. The in-plane strain/stress values computed from these slopes using equations (8*c* [Disp-formula fd8c]) and (8*e* [Disp-formula fd8e]) were identical to the predictions of the analytical computations [equations (13*a* [Disp-formula fd13a]), (13*b* [Disp-formula fd13b]) and (14)] and to the direct-space results from the FEM simulation. Since our numerical results agreed completely with the isotropic linear elasticity analysis, we concluded that our model was mathematically sound. We then extended this approach to model the strain/stress distribution in an ideal Cu slab by substituting Cu stiffness parameters and the Cu CTE in the model definition; all other parameters were unchanged.

(*b*) *Single-phase Cu slabs.* In the Cu model the distribution of elastic moduli in the sample coordinates is heterogeneous. Thus, the constraint stresses imposed by the fixed boundaries in the plane of the slab during heating will cause Heyn strains/stresses to arise within the model. These local values were computed by FEM at the node scale. We used these local values to compute average stress/strain tensors for individual grains and for various volumes of interest. We also computed the expected stress/strain states in an equivalent isotropic Cu slab [equations (13*a* [Disp-formula fd13a]), (13*b* [Disp-formula fd13b]) and (14)] using macroscopic values for Cu Young’s modulus and Poisson’s ratio. In this model, to avoid plastic flow, we specified a 25°C temperature increase.

**Figure 7:**
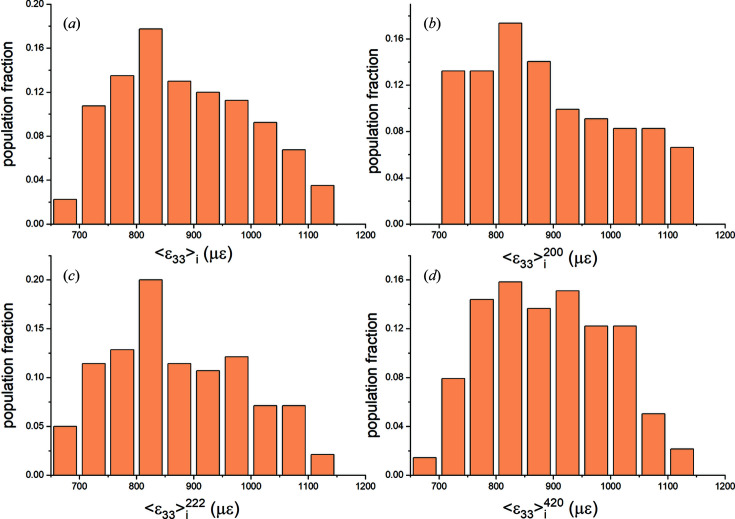
Distributions of direct-space out-of-plane grain-averaged normal strain 〈ɛ_33_〉_*i*_ within the plane-constrained Cu mesh subjected to a 25°C temperature increase. (*a*) The entire mesh, (*b*), (*c*) and (*d*) subset distributions for the 200, 222 and 420 reflection volumes, respectively.

*Direct-space analysis.* In Figs. 7 ▸(*a*)–7 ▸(*d*) the distributions of grain-averaged out-of-plane strains 〈ɛ_33_〉_*u*_ (*u* = 1 to *N*) for the entire mesh and for the particular reflection volumes are plotted. Strain components 〈ɛ_11_〉, 〈ɛ_22_〉 and 〈ɛ_12_〉 had similar distributions. Shear strains 〈ɛ_13_〉 and 〈ɛ_23_〉 were negligible. Average values and dispersion parameters for elastic strains 〈ɛ_*ij*_〉 are listed in the top half of Table 6 ▸. The mesh and reflection averages of all strain components are within 50 µɛ of the values predicted for an equivalent isotropic Cu slab.

For most strain distributions Shapiro–Wilk and Kolmogorov–Smirnov tests could not reject normality at the 0.05 level (Razali & Wah, 2011 ▸). Thus, in general, the Heyn interaction strains are normally distributed. This was expected: each 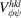 has a finite number of grains 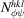 and each grain is modelled with a very large number of nodes. Thus the strain within the *n*th grain will be a mean value 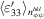 taken over all these nodes, with a deviation 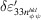 . The grain-averaged strains 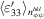 are then averaged again to obtain the ψ volume average 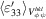 . Thus, in the absence of *systematic* partitioning of strains between grains in a ψ volume, the central limit theorem predicts that the distribution of 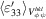 must be Gaussian if enough nodes and enough grains are sampled (Fox, 2015 ▸). In the bottom half of Table 6 ▸ the stress distribution parameters are listed. The stress and strain distributions were very similar.

**Figure 8:**
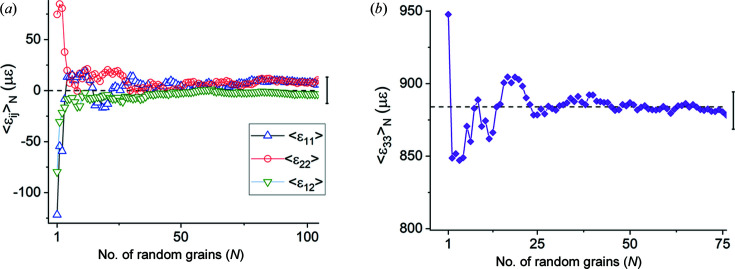
Cumulative direct-space averages of strain components (*a*) 〈ɛ_11_〉_*N*_, 〈ɛ_12_〉_*N*_ and 〈ɛ_22_〉_*N*_, and (*b*) 〈ɛ_33_〉_*N*_ with number of grains *N*, randomly selected from the constrained Cu mesh heated by 25°C. The horizontal dashed lines show the overall mesh averages. The vertical error bars on the far right in each plot span one standard deviation

The two halves of Table 6 ▸ show that in direct space all three reflection volumes are, individually, larger than RVE_DS_ for this model since they yield average values equal to the mesh averages. To determine the minimum number of grains constituting an RVE_DS_ we used a random number generator to select grains while monitoring the (running) average strain and stress values of the cumulative ensemble. Results for selected strains are shown in Figs. 8 ▸(*a*) and 8 ▸(*b*). Between 30 and 40 grains are sufficient to obtain the mesh average within one standard deviation; this number is comparable to our experimental results for plastic strains in Pb–Sn solders (Bonda & Noyan, 1992 ▸, 1996 ▸).

**Figure 9:**
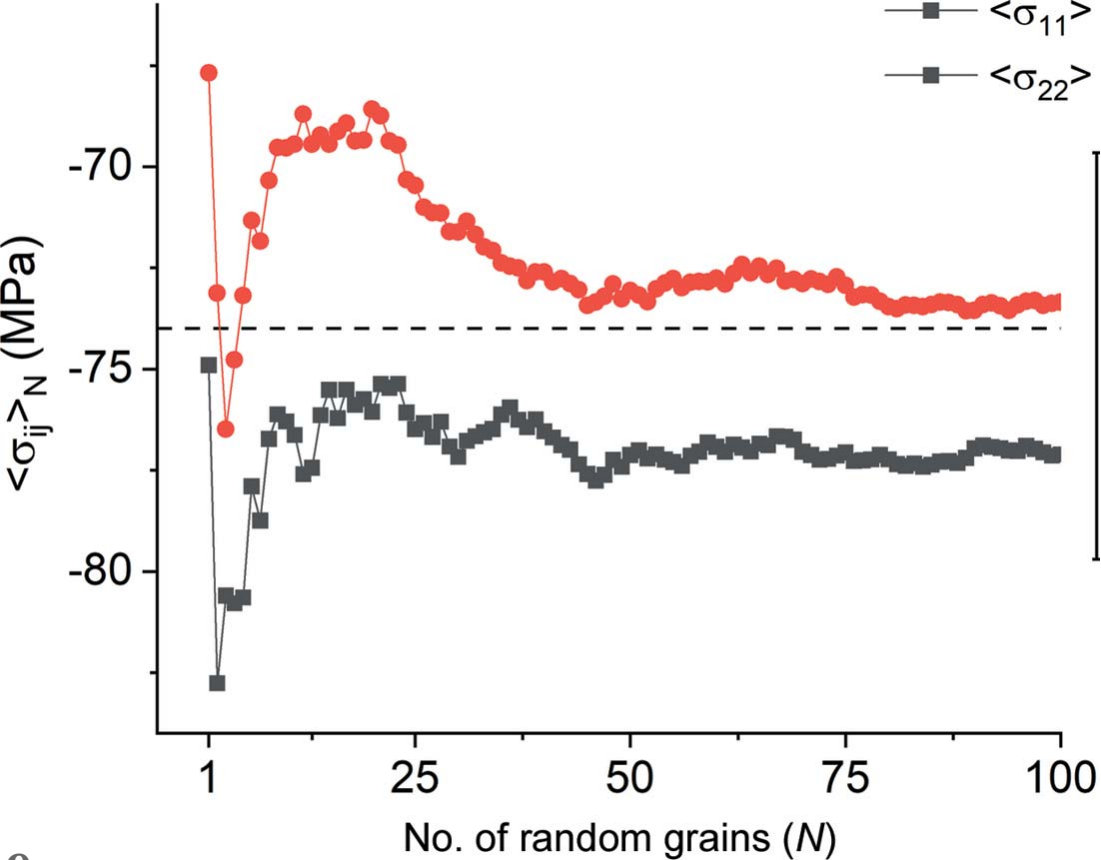
Cumulative direct-space average in-plane normal stresses with number of grains randomly selected from the constrained Cu mesh heated by 25°C. The horizontal dashed line shows the expected stress for an equivalent isotropic slab. The vertical error bar on the far right spans one standard deviation.

The cumulative averages of the (finite) stress components exhibit similar behaviour (Fig. 9 ▸). Thus, for the current model, any direct-space RVE needs to contain approximately 35 grains in the plane of the sample. Since our model contains 400 grains, it is approximately one order of magnitude larger than RVE_DS_.

*Diffraction analysis.* Comparing Tables 2 ▸ and 6 ▸ we observe that, while all three reflection volumes are significantly larger than the direct-space RVE, individual ψ volumes for each reflection are smaller, each having, on average, half of the grains needed for RVE_DS_. To investigate how this deficiency *N*_ψ_ < *N*_RVE-DS_ impacted the diffraction stress results, we simulated the 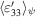 versus 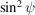 plots for all three reflections and used the standard 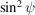 analysis based on equation (8*e* [Disp-formula fd8e]) to compute the in-plane stresses in the sample coordinates.

**Figure 10:**
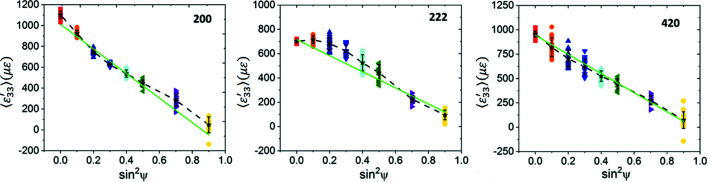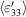 versus 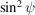 plots for the 200, 222 and 420 reflections for the in-plane constrained single-phase Cu mesh heated by 25°C. At each ψ angle, strain values for all diffracting grains are shown. For all plots the dashed line connects the average strain values (included to guide the eye). The green solid straight lines depict least-squares fits.

The computed 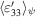 versus 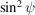 plots are shown in Fig. 10 ▸. Both the 200 and 222 reflections exhibit slight deviations from linearity. These deviations are more pronounced for the 222 reflection. We used linear regression to fit straight lines to these plots (solid green lines) and utilized the fitted line slopes with the Cu Neerfeld–Hill and Kröner diffraction moduli (Table 4 ▸) to obtain the in-plane stresses. These results are shown in Table 7 ▸, along with the stress values computed for the isotropic equivalent slab using equation (14) for comparison. We observe reasonable agreement within statistical uncertainty, indicating that, even when each ψ volume is somewhat smaller than the direct-space RVE, the overall stress value computed by analysing all the ψ volumes together is acceptable if (i) the total reflection volume is much greater than the RVE and (ii) the distribution of Heyn stresses is random in all grains of all ψ volumes. The bottom half of Table 6 ▸ also shows that the ‘fit errors’ of the computed stresses obtained from regression analysis do not adequately reflect the direct-space dispersion of direct-space stress values.

The closeness of the stress values computed from equation (14) to the stresses obtained from simulated diffraction analysis also indicates that our model is a good approximation of a random polycrystalline slab. Consequently, formulations based on texture-induced elastic anisotropy in thin-film geometries, such as the Vook–Witt or inverse Vook–Witt approaches (Vook & Witt, 1965 ▸; Witt & Vook, 1968 ▸; Welzel & Fréour, 2007 ▸; Welzel *et al.*, 2006 ▸), are not applicable for our models.

(*c*) *Effects of plastic deformation in the single-phase Cu model.* Here the (undeformed) Cu mesh was subjected to a 70°C temperature increase. The total stresses caused by the constrained in-plane thermal expansion induced (heterogeneous) plastic flow in all grains.

**Figure 11:**
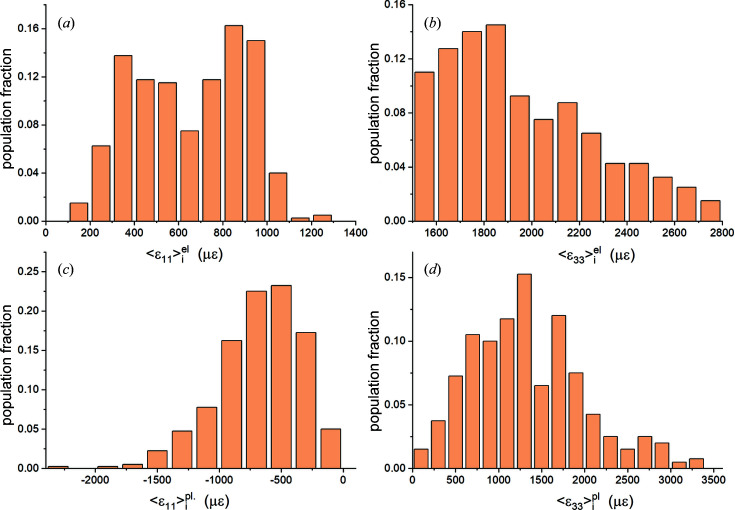
Distributions of in-plane and out-of plane direct-space grain-averaged strains 〈ɛ_11_〉_*i*_ and 〈ɛ_33_〉_*i*_ within the entire Cu mesh when the temperature is increased by 70°C. (*a*), (*b*) Elastic strain components and (*c*), (*d*) plastic strain components.

**Figure 12:**
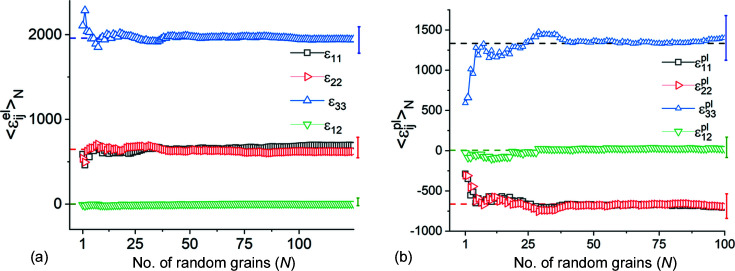
The variation in cumulative average (*a*) elastic and (*b*) plastic strains with number of grains randomly selected from the edge-constrained polycrystalline Cu model for Δ*T* = 70°C. The vertical error bars on the far right span one standard deviation (±½STD).

*Direct-space analysis.* Table 8 ▸ lists the direct-space mesh averages and their dispersion parameters for plastic strains, elastic strains and stresses. While the Cu slab is still subjected to far-field isotropic compressive plane stresses due to boundary constraints, the average stress magnitude in the slab is approximately half of the value predicted by fully elastic solutions [equation (14)] due to plastic flow. The mesh-averaged plastic and elastic strains are comparable in magnitude, with plastic strains having much wider distributions. The shapes of these strain distributions, however, are different: while normality could not be rejected for plastic strain distributions, elastic strain distributions failed both normality tests (Fig. 11 ▸). On the other hand, the RVE_DS_ values obtained for elastic or plastic strains (Fig. 12 ▸) are very close (∼35 grains) and are similar to the RVE_DS_ obtained for the elastic loading of the Cu mesh, indicating that plastic flow of this magnitude (≲0.3%) does not significantly impact the number of grains constituting a representative volume element for single-phase Cu.

**Figure 13:**
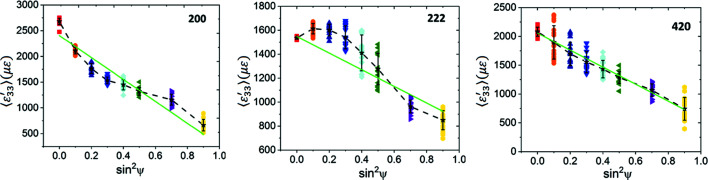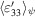 versus 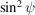 plots for the 200, 222 and 420 reflections for the in-plane constrained single-phase Cu mesh heated by 70°C. At each ψ angle, strain values for all diffracting grains are plotted. The dashed lines connect average strain values (included to guide the eye). The green solid straight lines depict least-squares fits.

*Diffraction analysis.* 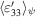 versus 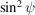 plots computed for this model are shown in Fig. 13 ▸ for all three reflections. The plots for the 200 and 222 reflections exhibit significantly larger nonlinearities than the elastic model, even though the overall forms are similar. These oscillations cannot be attributed to changes in texture or elastic anisotropy, since these parameters are identical to their values for the fully elastic case. The deviations from linearity are also much larger than any statistical (random) scatter expected in the data; the average strain components for many ψ volumes are further out from the regression fit line than the full data spread for the particular ψ volume. We note that elastic anisotropy should not have caused oscillations in the 200 and 222 reflections if the global Reuss assumption were valid (Noyan & Cohen, 1987 ▸).

Table 9 ▸ shows that the in-plane stress values computed from linear regression analysis of these plots using the Neerfeld–Hill and Kröner diffraction moduli do not agree with either the overall mesh average or the direct-space average stress components for the grain subsets contributing to these reflections;^2^ stresses computed with DEC values at the Voigt and Reuss limits would have yielded much larger errors. We also observe that linearity of a 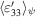 versus 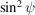 plot does not guarantee accuracy; the most ‘linear’ plot, corresponding to the 420 reflection, which has the smallest ‘fit’ errors, exhibits similar stress differences from the mesh average to the nonlinear (‘oscillatory’) plots for the 200 and 222 reflections.

We conclude that, for single-phase non-textured polycrystals, non-random distribution of Heyn stresses in diffraction volumes is the most probable cause of systematic errors in diffraction stress analysis, since this is the only difference between our elastic and elasto-plastic Cu models. To investigate further the effects of systematic partitioning of such strains and stresses among diffraction volumes, we modelled the free thermal expansion of a W–Cu slab heated to Δ*T* = 70°C. Here the mutual constraint between Cu and W grains is expected to cause residual stresses balanced between the two phases. In this exercise we also checked whether elastic anisotropy of the constituent crystallites is the main driver of oscillations. If this were the case, direct-space and diffraction average stress values should be comparable.

### 5.2. Unconstrained thermal expansion in a two-phase (Cu–W) slab model

*Direct-space analysis.* This model consisted of 676 hexagonal grains in a single-layer 26×26 matrix, with 352 elasto-plastic Cu grains and 324 fully elastic W grains randomly placed in the mesh and connected to each other across rigid boundaries normal to the slab plane. The slab boundaries were free to expand in all three directions. This model was subjected to a uniform temperature increase of Δ*T* = 70°C, and relevant stress and strain parameters were obtained from the (converged) finite-element solution. In this model plastic flow was confined to the softer Cu phase. This plastic strain distribution is summarized in Table 10 ▸. Most of the plastic flow is accommodated by in-plane shear strains.

Table 11 ▸ lists the direct-space elastic strains and stresses averaged over the entire mesh and its two phases. These residual stresses formed in response to the systematic partitioning of plastic flow between grains of the two phases. As required by the (unconstrained) far-field slab boundary conditions, all average stress tensor components for the complete mesh are zero. The average normal stresses 〈σ_*ii*_〉 and the in-plane shear stress 〈σ_12_〉 have very broad distributions. The magnitudes of the out-of-plane shear stresses/strains 〈σ_*j*3_〉 and 〈ɛ_*j*3_〉 (*j* = 1, 2) are very close to zero with very narrow distributions; these terms are neglected in the following discussion.

**Figure 14:**
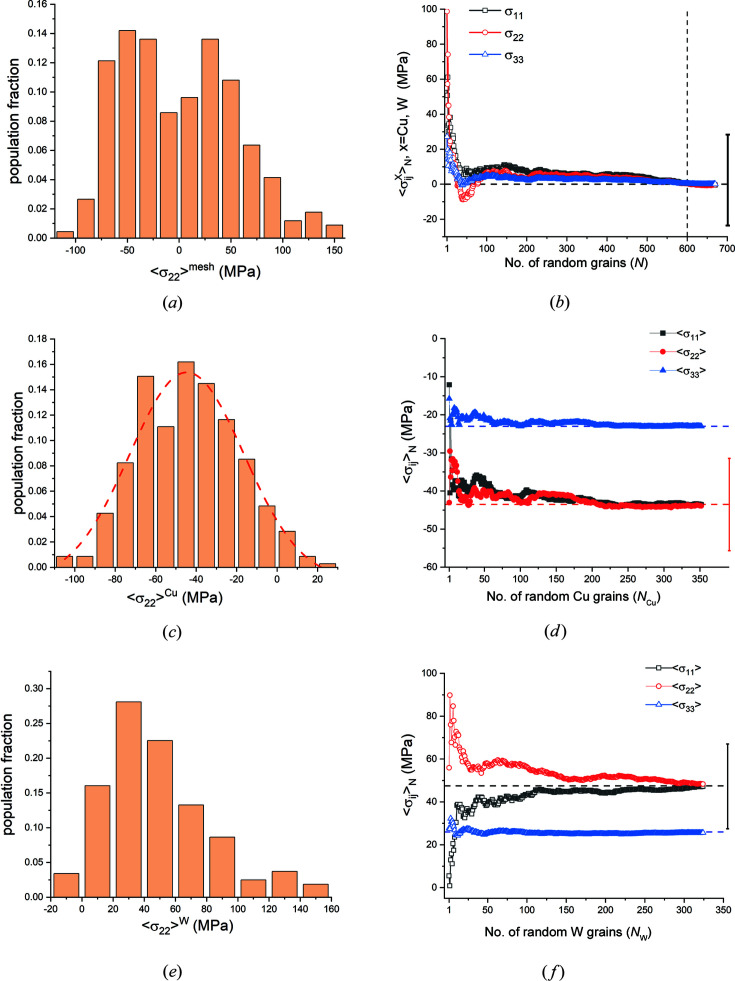
Distributions of the in-plane grain-averaged stress component 〈σ_22_〉 for (*a*) the entire Cu–W model and (*c*), (*e*) its constituent phases, Cu and W, respectively. The red dashed line in panel (*c*) is a Gaussian regression fit, included to guide the eye. (*b*), (*d*), (*f*) The corresponding representative volume element analysis results. In these plots the solid bars on the far right depict one standard deviation of 〈σ_22_〉 after the running average has stabilized.

The distributions for the average stress and strain components over the entire mesh are not Gaussian. A representative example is shown in Fig. 14 ▸(*a*). RVE analysis showed that ∼500 randomly picked grains are needed before the cumulative stress averages converge to the mesh averages [Fig. 14 ▸(*b*)]. This is more than an order of magnitude larger than the RVE for the single-phase Cu model. In direct space the stress and elastic strain distributions for the Cu phase (which suffered plastic flow) are close to normal (Gaussian) distributions [Fig. 14 ▸(*c*)]. The corresponding terms for the (fully elastic) W phase are not normally distributed [Fig. 14 ▸(*e*)]. RVE analysis showed that approximately 200 and 300 grains are required for the in-plane stress averages to reach the phase-averaged stress values for the Cu and W phases, respectively [Figs. 14 ▸(*d*) and 14 ▸(*f*)]. Since W is isotropic, the broad stress/strain distributions in the W phase are caused solely by local constraint of the Cu phase.

The stress values in Table 11 ▸ show that, in contrast to the single-phase models, where the mesh was under plane stress, the average stress tensors for the Cu and W phases are triaxial,^3^ with all normal stress components having the same sign for a given phase (compressive and tensile, respectively). Further, all average stress components 〈σ_*ii*_〉_*t*_ (*t* = Cu, W) satisfy the equilibrium condition for average phase stresses (Noyan, 1983*b* ▸),

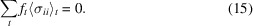

Here the average shear stresses normal to the surface, 〈σ_*i*3_〉_*t*_ (*i* = 1, 2), are not included in the summation due to their negligible magnitudes.

In summary, direct-space analysis of the stresses within the grains of the two-phase model showed that while, as dictated by the conditions of equilibrium, the overall stress is zero, individual phases have triaxial average stress states which balance each other. Given the distributions in Fig. 14 ▸, average stresses are quite inadequate to capture the stress state of the overall system or the distribution of local stresses. Consequently, direct-space free-body diagrams which contain material volumes ≥ RVE_DS_ are inadequate to represent the internal stress distributions within such systems.

**Figure 15:**
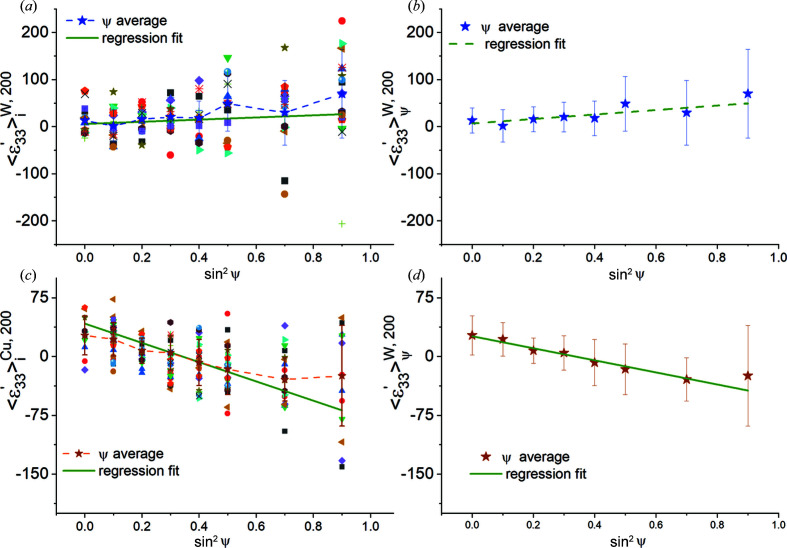
The variation in grain-averaged strains 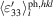 with 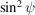 for the 200 reflections of (*a*) W and (*c*) Cu in the W–Cu composite subjected to a 70°C temperature increase. The dashed lines in these plots connect the average strain values and are included to guide the eye. Solid lines are from linear regression analysis. (*b*) and (*d*) Plots of only ψ volume averaged strains 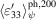 . Solid lines are from linear regression analysis for these average values.

*Diffraction analysis*. The variation in grain-averaged strains 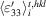 (*t* = Cu, W) with 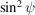 for the 200 reflections of W and Cu is shown in Figs. 15 ▸(*a*) and 15 ▸(*c*), respectively. The solid lines in these plots show the linear regression fits, while the dashed lines connect the average strain values at each ψ and are included to guide the eye. In Figs. 15 ▸(*b*) and 15 ▸(*d*), ψ volume averaged strains 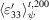 versus 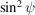 and linear fits to these average values are plotted; the error bars span one standard deviation. We note that, for both phases, the ψ-averaged data, which are the quantities measured by diffraction, do not adequately reflect the dispersion of the individual grain-averaged strains.

Taken together, Figs. 14 ▸ and 15 ▸ show that neither the distribution breadths of grain-averaged strains along various directions nor the ‘linearities’ of 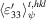 versus 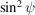 plots are strongly correlated with the Zener indices *Z*_I_ of the phases from which they originate. The linearities of 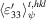 versus 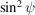 (*t* = Cu, W) plots were comparable for all modelled reflections. In addition, deviations of the average strain values from their real-space counterparts were not correlated with either the indices of particular reflections or the *Z*_I_ of the particular phase.

Table 12 ▸ lists the slopes and intercepts obtained from linear regression analysis of the average grain data for both phases 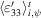 versus 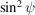 (*t* = W, Cu). For the 200 reflections of both phases, slopes and intercepts obtained from fitting the ψ-averaged strain data [Figs. 15 ▸(*b*) and 15 ▸(*d*)] are also reported. The values in parentheses for 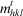 and 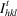 correspond to standard errors reported by the regression fitting program. For the 200 reflections, using the average strain data for each ψ volume significantly reduces the standard errors associated with 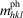 and 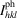 . In the last two columns of Table 12 ▸ we list the stress values 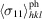 and 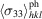 calculated from equation (8*c* [Disp-formula fd8c]) using DEC values at the Kröner limit. The tabulated stress uncertainties were obtained by error propagation.

The (direct-space) reflection-average stresses obtained directly from the finite-element model (Table 11 ▸) and from the simulated diffraction analysis (Table 12 ▸, last two columns) show agreement in the *sign* of the stress values for both phases: both techniques yield compressive residual stresses for the Cu phase and tensile stresses for the W phase. The computed stress magnitudes, on the other hand, show deviations – most average diffraction stress values are about half of the corresponding real-space averages. The discrepancies between direct-space and diffraction-based stress values could have been much worse: under normal experimental circumstances one would have used the biaxial 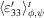 versus 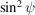 equation [equation (8*e* [Disp-formula fd8e])] for analysis, not the triaxial one [equation (8*c* [Disp-formula fd8c])], since the slab is only one grain thick. For such a case the stress components 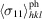 computed using only the slopes would have had deviations from the direct-space values equal in magnitude to the 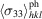 components (Noyan & Cohen, 1987 ▸).

The uncertainty values of the diffraction-based stress results obtained by fitting the ψ average strain data 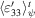 (*t* = W, Cu) are much smaller than those obtained from fitting the individual grain values (Fig. 15 ▸). Since, in actual experiments, only ψ-averaged strain data are available, regression analysis underestimates the uncertainty associated with the computed stress values. Finally, even though the ψ-averaged phase strain 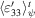 data shown in Figs. 15 ▸(*b*) and 15 ▸(*d*) seem ‘linear’, omission of a few data points can yield significantly different slopes and intercepts. For example, if only the first five points in the W plots [Figs. 15 ▸(*a*) and 15 ▸(*b*)] were used, the slope would have been close to zero, yielding a very low stress value. Thus, these plots should not be considered ‘linear’. We conclude that the elastic anisotropy of the unit cell is not always the sole cause, or even the primary cause, of non­linearities in 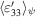 versus 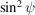 data.

## 6. Discussion

The basic theoretical development [equations (1*a* [Disp-formula fd1a])–(12)] shows that, within an ROI of a statistically homogeneous untextured polycrystalline body, the equivalence of an elastic (Cauchy) stress tensor measured with mechanical techniques and the tensor measured by diffraction is possible if and only if both sampling adequacy and strain uniformity conditions are satisfied:

(i) Sampling adequacy condition: measurement domain(s) utilized for both techniques are representative elements of the entire ROI with respect to physical properties.

(ii) Stress uniformity condition: the total volumes of these measurement domains for each technique, respectively, can be represented by free-body elements with identical uniform traction distributions on their boundaries.

(Both of these conditions are trivially satisfied in homogeneous solid continua loaded uniformly at the far field.)

Even when these conditions are satisfied, the requirements for representative volume elements are different for direct-space and diffraction techniques. The average elastic moduli 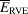 and 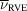 , and stress 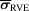 and strain 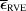 values for any direct-space RVE_DS_ must be identical to their corresponding values for a material point within an equivalent isotropic solid loaded at the far field with stress tensor **σ** ^0^. For diffraction measurements based on equations (8*a* [Disp-formula fd8a])–(8*e* [Disp-formula fd8b] [Disp-formula fd8c] [Disp-formula fd8d] [Disp-formula fd8e]), all subsets of the reflection volume *V* ^*hkl*^ utilized in an experiment must possess homogeneous distributions of the average elastic moduli and stress and strain tensors. In addition, if *V* ^*hkl*^ is to be considered a diffraction representative volume 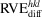 , the average diffraction stress tensor, computed with Neerfeld–Hill or Kröner DEC, must be equal to the far-field stress tensor: 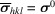 , *i.e.* the average of all Heyn stresses must be statistically equal to zero within the fibre-textured discontinuous volume 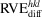 .

To investigate the effects of elastic and plastic strain distributions in a set of fully coupled anisotropic crystallites on the relevant representative volume elements we used FEM analysis. Our first three models contained isotropic stress and strain distributions. For single-phase Cu and W polycrystalline slab models (Models 1 and 2, respectively) subjected to unconstrained thermal expansion and a W polycrystalline slab subjected to constrained thermal expansion (Model 3), the direct-space strain/stress tensors computed at finite-element nodes or elements, or averaged over various material volumes, were identical. The 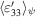 versus 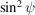 plots computed from diffracting grains at the modelled ψ angles were exactly linear and yielded strain/stress values identical to the direct-space analysis.

A polycrystalline Cu slab subjected to constrained thermal expansion was studied in Model 4. In this case local stresses/strains developed to keep the grain boundaries compatible in response to the uniform stress exerted at the (far-field) boundary. These Heyn stresses/strains were approximately randomly distributed within the model and were self-balancing: they averaged out to zero over the entire mesh, and over representative volume elements. A simple statistical analysis showed that a minimum of approximately 35 grains (∼10% of the mesh population) were required in direct space for the interaction stresses/strains to average out. Thus, all volumes containing 35 grains or more could be considered direct-space representative volume elements and replaced by a material point in an equivalent isotropic Cu slab.

The presence of Heyn strains caused slight deviations from linearity in 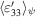 versus 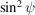 plots for all reflections modelled in the analysis, since the ψ volumes over which strains were averaged at each tilt angle were smaller than the RVE required to average out the interaction strain components. However, since the distributions of the interaction strains were random with tilt angle, linear regression analysis based on a biaxial stress state model [equation (8*e* [Disp-formula fd8e])] yielded average in-plane stress values acceptably close to those computed for an equivalent isotropic Cu slab heated by 25°C with in-plane boundary constraint. We note here that, in minimizing deviations from the model, simple linear regression of 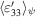 versus 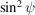 plots assumes implicitly that all ψ volumes are of similar magnitude and contain similar numbers of similarly sized grains. Thus, all data points (and their errors as applicable) are given equal weight. This was the case for our model. If some ψ volumes are much smaller than others (due to texture or a grain size distribution with long tails) their deviation from linearity in response to a far-field traction might be large. In such cases a weighted regression model might be used. However, it might not be justified to use Bragg peak intensities as weights in this case. If there is a distribution of grain sizes in the diffracting volume, the integrated or maximum peak intensities might not accurately reflect the volume fraction of diffracting grains (Noyan & Kaldor, 2004 ▸).

In our next model (Model 5), the same Cu mesh as utilized in Models 2 and 4 was heated under in-plane boundary constraint to Δ*T* = 70°C. This caused plastic flow in all Cu grains, with compressive in-plane plastic strains. Plastic strains normal to the slab plane were tensile. This level of plastic flow did not change the number of grains constituting a direct-space RVE; averaging over 35 to 40 random grains was sufficient to reach the mesh averages of elastic and plastic strain, as well as of stress components. The direct-space mesh-averaged in-plane stress components 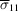 and 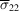 were approximately half of the stress expected for a fully elastic model due to plastic relaxation. The out-of plane stress component 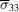 was negligible. The direct-space stress averages of individual reflection volumes were very close to the overall mesh averages. In this model, while plastic strains exhibited quasi-Gaussian distributions, the distributions of the elastic strains in the Cu grains were no longer Gaussian.

For this model, 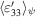 versus 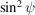 plots for all reflections exhibited statistically significant nonlinearities. Diffraction stress analysis using simple linear regression of the 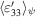 versus 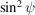 plots yielded stress values which deviated by more than one standard deviation from the direct-space stress averages of the corresponding reflection volumes. Arithmetic averages of the stress values for the three reflections, which constitute almost the entire mesh volume, did not show better agreement. The only difference between this Cu model and Models 2 and 3 (free expansion of Cu mesh, and biaxial elastic loading of Cu mesh by uniform edge constraint, respectively; all three models were based on the same mesh, with identical grain locations, shapes and orientations) is the non-random distribution of Heyn interaction strains in the Cu grains due to plastic flow. We conclude that, if the distribution of elastic strains in the constituent grains is non-random, even a ‘linear-seeming’ 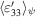 versus 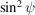 plot of a particular reflection can yield stress values which are not equal to the direct-space far-field loads or to the (arithmetic) average stress components in the corresponding reflection volumes. This is expected, since regression analysis assumes that the means of (random) errors are zero and no systematic errors are present (Fox, 2015 ▸; Seber & Wild, 2003 ▸; Seber, 2015 ▸).

Our final model (Model 6) consisted of heating a two-phase Cu–W slab with free boundaries to Δ*T* = 70°C. In this case there were no boundary (far-field) stresses. There were, however, significant heterogeneous stress and strain fields within the slab caused by the mutual constraint of Cu and W grains arising from their different CTEs; the W grains constrained the Cu grains from achieving their free-body dimensions at the higher temperature and placed them in compression, while the Cu grains pulled on the less-expanding W grains and placed them in tension. The degree of compression caused plastic flow in Cu, while the W grains were fully elastic. In this system the direct-space RVEs required approximately ten times more grains than the single-phase models. Diffraction stress analysis using simulated 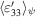 versus 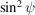 plots yielded stress values with the correct sign for all reflections of both phases. The stress magnitudes, however, did not show good agreement with the direct-space averages, similar to the case for Model 5 where plastic flow was also finite.

For this two-phase model, the phase-average stresses obtained from diffraction analysis could not be assigned to direct-space ‘equivalent isotropic volumes’ of the corresponding phases due to the broad stress/elastic strain distributions. In fact, one could not even predict the (average) stress state within a given grain of a particular phase from the phase-average stresses: in our model some grains in Cu had tensile stresses and some in the W phase had compressive stresses.

Mechanical methods such as hole drilling or sectioning, which sample volumes ≥ RVE_DS_, will yield a zero stress field in such (two-phase) samples since they lack phase specificity. However, such a result does not represent the true stress distribution within the material. For such samples it would be better to use both mechanical and diffraction methods.

## 7. Summary and conclusions

Our results show that the main difference in measuring residual stresses using mechanical or single-reflection-based diffraction methods stems from the very different volumes sampled, and averaged over, by the respective probes. In almost all cases, both the geometry and the topology of the sampled volumes are different. The diffraction techniques take multiple averages at each stage of the measurement, with each average assigning different ‘weights’ to the averaged parameters. First, a Bragg peak at a given ψ tilt yields the average lattice parameter, and hence strain, over those grains oriented to diffract at the particular ψ angle for the particular reflection; the strains within each such grain are also averages over the respective grain volume. Second, when one then uses classical linear regression to refine the strain tensor employing the average strains referred to the ψ volumes utilized in the measurement, one usually assigns equivalent weights to all strain values, intimating that all ψ volumes contain comparable grain populations. This final ‘reflection average’ originates from only a fraction of the illuminated volume. Thus, the average stress tensor computed from a single reflection average may or may not be equal to the far-field stress tensor of the ROI. This depends on the partitioning of elastic strains between different texture groups. Asserting, without proof, that the measured 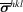 is identical to the far-field stress **σ** ^0^ at ROI boundaries might lead to wrong conclusions.

**Figure 16:**
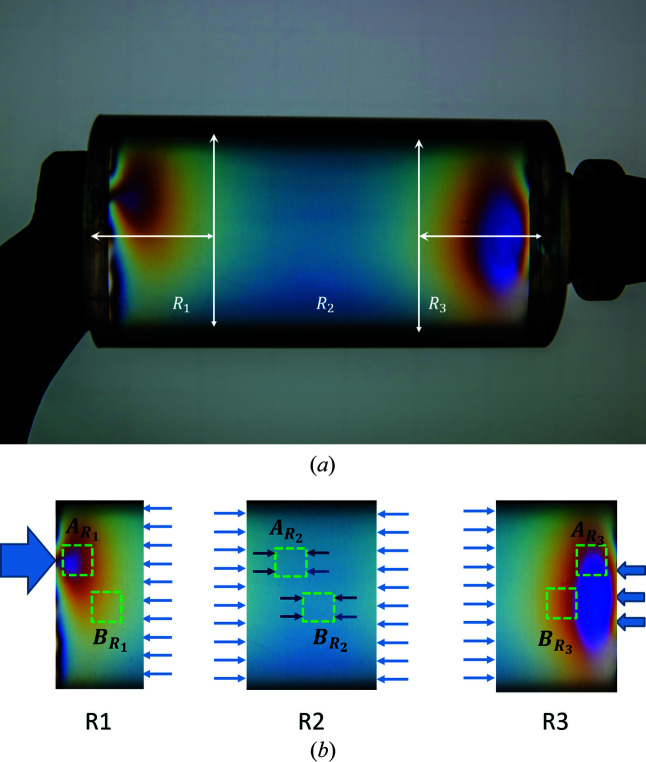
(*a*) Isochromatic contours in a homogeneous acrylic cylinder compressed by a C-clamp. Regions *R*_1_ and *R*_3_ have Saint-Venant stresses and strains due to imperfections in the clamp surfaces. Region *R*_2_ has a homogeneous stress/strain distribution. (*b*) Idealized free-body diagrams of these regions, each with two regions of interest (green dashed squares). While one can infer the boundary stresses acting on the free-body diagrams of 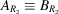 from nominal stresses at the cylinder boundary, this is not possible for those in *R*_1_ and *R*_3_.

In practice, most diffraction strain experiments are time constrained and aim to use the fewest possible measurements to obtain a usable result. If the goal is to identify Saint-Venant regions or other ROIs where rapid changes in stress/strain occur, this can be accomplished by simply mapping the average diffraction strain 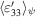 at a particular ψ tilt for a number of direct-space locations within the particular ROI before undertaking a full stress measurement. For ROIs with homogeneous strain distributions, such as regions *A*_*R*2_ and *B*_*R*2_ in Fig. 16(*b*) (Appendix *B* [App appb]), 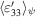 will be (statistically) independent of position. If this is not the case, one can then adjust the X-ray spot size to achieve homogeneity. Such strain maps, when combined with metallographic grain size analysis and texture measurements, can be used to guide further stress measurements. In contrast to stress analysis, strain values obtained by diffraction can be very accurate and precise (Noyan *et al.*, 2020 ▸) since, for strain mapping, one does not need to combine interpenetrating volumes for the tensor transformations required for stress analysis.

If the goal is to obtain residual stress values which are identical to those from mechanical measurements, one must ensure that diffraction strains are measured over a representative volume of the entire ROI. This requires measurement of multiple reflections, each with as many ψ tilts as practically feasible. If the measured 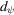 versus 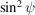 or 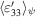 versus 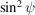 plots are linear (regular) for all reflections, and yield the same stress value with appropriate DEC, there is at least some indication that the sampled reflection volumes represent a direct-space RVE. This equality is a necessary but insufficient condition. Such validation is especially important in samples where systematic partitioning of elastic (and plastic) strains is possible (*e.g.* samples subjected to directional deformation, multi-phase samples or textured thin films). If systematic deviations from linearity are observed for any reflection, diffraction analysis must be supplemented by further analysis, including metallography, texture measurements, polycrystalline finite-element models and mechanical (relaxation) measurements. Curve fitting of diffraction data using formalisms based solely on elastic anisotropy should be avoided; elastic anisotropy of the unit cell is not always the sole cause, or even the primary cause, of nonlinearities in 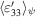 versus 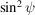 data.

## Figures and Tables

**Table 1:** Zener indices *Z*_I_, stiffness matrix components *C*_*ij*_, average elastic moduli 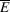 and 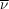 , and coefficients of thermal expansion for the materials used in our FEM models *C*_*ij*_ are in units of GPa, 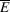 in GPa and CTE values in 1/°C. F.c.c denotes face-centred cubic and b.c.c. denotes body-centred cubic.

Material	Structure	*Z*_I_	*C*_11_	*C*_12_	*C*_44_	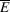	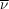	CTE × 10^6^
Cu	F.c.c.	3.20	168.4	121.4	75.4	112	0.34	16.7
W	B.c.c.	1.00	501	198	151.4	385	0.27	4.6

**Table 2:** ψ ensemble populations 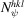 and volume fractions 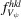 for the three reflections in the mesh Total reflection ensemble populations *N* ^*hkl*^ and their (number) fractions *f* ^*hkl*^ are given in the last column.

	ψ angle (°)								Totals *N* ^*hkl*^, *f* ^*hkl*^
	0	18.43	26.57	33.21	39.23	45	56.79	71.57	
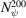	16	16	14	14	15	17	17	12	121
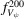	0.040	0.040	0.035	0.035	0.038	0.043	0.043	0.030	0.303
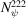	18	18	18	17	16	18	18	17	140
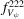	0.045	0.045	0.045	0.043	0.040	0.045	0.045	0.043	0.35
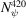	18	18	17	16	18	18	17	17	139
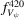	0.045	0.045	0.043	0.040	0.045	0.045	0.043	0.043	0.348

**Table 3:** Diffraction elastic constants (TPa^−1^) of W calculated from single-crystal stiffness values (Table 1 ▸) using various approaches; these values are independent of reflection since W crystals are isotropic in elastic loading

	Reuss	Voigt	Kröner	Neerfeld–Hill
−ν/*E*	0.70	0.70	0.70	0.70
(1 + ν)/*E*	3.30	3.30	3.30	3.30

**Table 4:** Diffraction elastic constants (TPa^−1^) for an untextured Cu polycrystalline sample, calculated from single-crystal stiffness values at various limits

	*hkl*	Reuss	Voigt	Kröner	Neerfeld–Hill
−ν/*E*	200	6.28	2.24	3.73	4.26
−ν/*E*	222	1.40	2.24	1.93	1.82
−ν/*E*	420	3.94	2.24	2.87	3.09
(1 + ν)/*E*	200	21.28	9.17	13.63	15.23
(1 + ν)/*E*	222	6.65	9.17	8.24	7.86
(1 + ν)/*E*	420	14.26	9.17	11.04	11.73

**Table 5:** Stress and strain tensors for the edge-constrained W thin-slab model heated to 70°C, obtained from analytical formulations [equations (13*a* [Disp-formula fd13a]), (13*b* [Disp-formula fd13b]) and (14)], and direct-space and diffraction averages computed from FEM

Parameter	Equations (13*a* [Disp-formula fd13a]), (13*b* [Disp-formula fd13b]) and (14)	*ABAQUS/CAE*	X-ray diffraction analysis
Thermal strain ɛ^Th,W^ (µɛ)	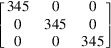	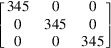	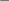
Boundary constraint strain, 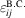 (µɛ)	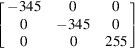	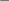	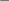
Total strain ( 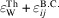 ); 〈ɛ_*ij*_〉 (µɛ)	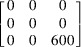	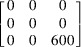	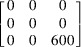
Stress σ_*ij*_; 〈σ_*ij*_〉 (MPa)	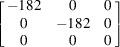	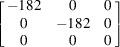	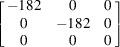

**Table:** Average values are highlighted in **bold**. SD denotes standard deviation. The ‘Range’ parameter encompasses the entire distribution breadth.

		Strain component (µɛ)					
Approach	Parameter	ɛ_11_	ɛ_22_	ɛ_33_	ɛ_12_	ɛ_13_	ɛ_23_
Isotropic slab	Equations (13*a* [Disp-formula fd13a]) and (13*b* [Disp-formula fd13b])	**0**	**0**	**848**	**0**	**0**	**0**
*ABAQUS/CAE*							
Full mesh (400 grains)	Average	**5**	**3**	**884**	**−3**	**0**	**0**
	SD	9	9	11	3	0	0
	Median	1	−4	872	−1	0	0
	Range	486	461	466	248	1	1
200 (121 grains)	Average	**44**	**−38**	**889**	**0**	**0**	**0**
	SD	9	8	12	3	0	0
	Median	52	−48	861	1	0	0
	Range	469	398	433	226	1	1
222 (140 grains)	Average	**−30**	**45**	**872**	**−5**	**0**	**0**
	SD	7	9	12	3	0	0
	Median	−37	59	858	−1	0	0
	Range	345	382	453	213	0	1
420 (139 grains)	Average	**5**	**−4**	**890**	**−5**	**0**	**0**
	SD	8	8	11	3	0	0
	Median	10	−4	888	−4	0	0
	Range	465	429	454	208	0	1

**Table:** 

		Stress component (MPa)					
Approach	Parameter	σ_11_	σ_22_	σ_33_	σ_12_	σ_13_	σ_23_
Isotropic slab	Equation (14)	−74	−74	0	0	0	0
*ABAQUS/CAE*							
Full mesh (400 grains)	Average	**−76**	**−74**	**0**	**0**	**0**	**0**
	SD	10	10	5	5	0	0
	Median	−76	−74	0	0	0	0
	Range	43	50	26	28	0	0
200 (121 grains)	Average	**−76**	**−74**	**0**	**0**	**0**	**0**
	SD	10	10	5	5	0	0
	Median	−76	−74	0	0	0	0
	Range	43	50	26	28	0	0
222 (140 grains)	Average	**−75**	**−78**	**1**	**−1**	**0**	**0**
	SD	9	10	5	5	0	0
	Median	−74	−78	0	0	0	0
	Range	38	47	24	23	0	0
420 (139 grains)	Average	**−76**	**−74**	**0**	**−1**	**0**	**0**
	SD	10	9	5	5	0	0
	Median	−76	−74	−1	−1	0	0
	Range	40	42	21	28	0	0

**Table 7:** In-plane average stresses for the boundary-constrained Cu mesh (Δ*T* = 25°C) obtained from slopes of regression-fitted lines to the data shown in Fig. 10 The stress values for the equivalent isotropic slab were computed using equation (14) with bulk elastic moduli, yielding σ_11_ = σ_22_ = −74 MPa.

	Diffraction analysis, 〈σ_11_〉^*hkl*^ (MPa)	
Reflection	At Neerfeld–Hill limit	At Kröner limit
200	−71 ± 4	−80 ± 5
222	−84 ± 8	−81 ± 7
420	−84 ± 3	−89 ± 3

**Table:** Average values are highlighted in **bold**. SD denotes standard deviation. The ‘Range’ parameter encompasses the entire distribution breadth.

			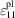	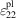	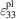	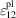	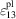	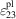
Plastic strain (µɛ)	FEM	Average	**−674**	**−659**	**1333**	6	0	0
		SD	344	350	655	231	0	2
		Median	−637	−610	1253	−11	0	0
		Range	2308	1886	3332	1979	3	10

**Table:** 

			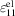	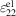	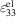	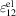	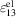	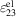
Elastic strain (µɛ)	FEM	Average	**653**	**640**	**1959**	0	0	0
		SD	249	226	312	34	0	0
		Median	682	653	1890	−1	0	0
		Range	1108	1146	1238	236	0	1
	Equations (13*a* [Disp-formula fd13a]) and (13*b* [Disp-formula fd13b])		0	0	2543	0	0	0

**Table:** 

			σ_11_	σ_22_	σ_33_	σ_12_	σ_13_	σ_23_
Stress (MPa)	FEM	Average	**−105**	**−105**	**−1**	0	0	0
		SD	10	9	4	5	0	0
		Median	−106	−106	0	0	0	0
		Range	54	54	22	28	0	0
	Equation (14)		−222	−222	0	0	0	0

**Table 9:** In-plane average stresses 〈σ_11_〉_*hkl*_ obtained from slopes of regression-fitted lines to the data shown in Fig. 13 with Neerfeld–Hill and Kröner diffraction moduli (Table 4 ▸) The standard errors shown in parentheses are ‘regression-fit’ errors. The FEM yielded σ_11_ = σ_22_ = −105 MPa.

	Diffraction analysis, 〈σ_11_〉^*hkl*^ (MPa)	
Reflection	At Neerfeld–Hill limit	At Kröner limit
200	−140 ± 20	−156 ± 22
222	−88 ± 17	−84 ± 17
420	−126 ± 4	−134 ± 5
Average of reflections	−118 ± 9	−125 ± 9

**Table 10:** Average plastic strain components (in microstrain) for the Cu phase of the two-phase model for Δ*T* = 70°C; there was no plastic flow in the W grains

Parameter	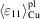	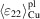	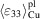	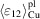	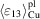	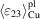
Average	−5	2	4	−14	3	1
Standard deviation	13	54	55	167	3	1
Maximum	84	187	365	798	15	4
Minimum	−58	−394	−222	−668	−1	−1

**Table 11:** Model parameters (number of grains *N*_*i*_ and volume fractions *f*_*i*_) and average elastic strain and stress components for the full model and its two phases Average stress values are highlighted in **bold**. SD denotes standard deviation. The ‘Range’ parameters show the total distribution breadth. Out-of plane shear components 〈ɛ_*i*3_〉 and 〈σ_*i*3_〉 (*i* ≠ 3) had negligible magnitudes and breadths; these are omitted for brevity.

		Strain (µɛ)				Stress (MPa)			
	Parameter	〈ɛ_11_〉	〈ɛ_22_〉	〈ɛ_33_〉	〈ɛ_12_〉	〈σ_11_〉	〈σ_22_〉	〈σ_33_〉	〈σ_12_〉
Full mesh, *N* = 676	Average	−57	−56	40	4	**0**	**0**	**0**	**0**
	SD	205	207	78	61	55	55	27	28
	Median	−12	−15	25	0	−11	-5	-3	
	Range	1340	1121	613	610	272	257	108	112

Cu phase, *N*_Cu_ = 352, *f*_Cu_ = 0.52	Average	−167	−173	72	−2	**−43**	**−44**	**−23**	**−1**
	SD	212	206	83	48	24	24	13	23
	Median	−166	−185	66	−1	−46	−44	−21	
	Range†	1340	1121	613	427	136	131	65	101

W phase, *N*_W_ = 324, *f*_W_ = 0.48	Average	63	75	−1	6	**47**	**48**	**26**	**1**
	SD	108	103	39	73	38	34	9	34
	Median	59	59	0	2	43	42	26	
	Range	651	519	208	610	212	173	52	203

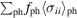	Equilibrium condition test for average stresses					**0.2**	**0.2**	**0.5**	

† The ranges for the entire mesh and for the Cu phase are equal for normal strains since the maximum and minimum strains occur in Cu grains.

**Table 12:** Slopes and intercepts [equation (8*e* [Disp-formula fd8e])] obtained from regression analysis, and reflection-average stresses computed from these values, for the Cu and W phases of the Cu–W slab heated by 70°C For the 200 reflection of both phases, regression results obtained from fitting the ψ average strain 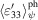 data are also shown. Standard errors reported by the regression program are shown in parentheses. The corresponding error values for the computed stresses were obtained by error propagation.

Phase (*t*)	Reflection	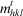 (µɛ)	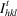 (µɛ)	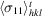 (MPa)	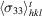 (MPa)
*t* = Cu	200 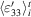	−123 (57)	42 (27)	−19 (13)	−10 (13)
	200 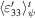	−77 (7)	26 (3)	−12 (2)	−7 (1)
	222 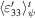	−49 (13)	4 (2)	−14 (2)	−8 (2)
	420 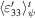	−61 (11)	4 (3)	−19 (2)	−14 (2)

*t* = W	200 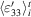	23 (93)	6 (45)	20 (50)	13 (41)
	200 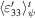	48 (15)	7 (5)	37 (7)	23 (5)
	222 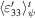	101 (21)	−10 (8)	58 (10)	27 (8)
	420 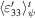	92 (14)	−1 (4)	60 (6)	32 (4)

## Footnotes

1 For an untextured polycrystal,  . The volume fractions of grains contributing to a given reflection can be computed from the classical Lorenz factor (Öztürk *et al.*, 2014 ▸).

2 For the 200, 222 and 420 reflection volumes, the direct-space in-plane average stress values 〈σ_11_〉_*hkl*_ ≅ 〈σ_22_〉_*hkl*_ computed from the FEM output were −105 ± 10, −105 ± 10 and −106 ± 9 MPa, respectively.

3 While the average in-plane stresses are equal in each phase, 〈σ_11_〉_*t*_ and 〈σ_22_〉_*t*_ (*t* = Cu, W), the average phase stresses 〈σ_33_〉_*t*_ along  are approximately half of the in-plane stress values. Since the film is unconstrained at both top and bottom surfaces (normal to  ), 〈σ_33_〉_*t*_ can only exist as a gradient along this axis, locally zero at each surface and locally balanced along the thickness by the variation in shear stress components.

## Appendix A Linear elasticity analysis

Linear elasticity theory is a special case of continuum-mechanics elasticity analysis, which provides a simple and useful mathematical model capable of describing fully reversible (elastic) deformation of a wide range of solid bodies under load. This approach is used extensively in structural analysis and engineering design; it is often implemented within numerical approaches such as finite-difference, finite-element or mean-field methods. In addition to being limited to elastic deformation, this approach assumes infinitesimal displacements and neglects rotations. These assumptions permit the use of linear (constitutive) relationships between the components of stress and strain tensors which describe the distribution of deformation and load within the solid volume.

Since almost all residual stress analysis formalisms utilize linear elasticity theory for computing residual stresses from (measured) displacements or elastic strain values, we include a very basic review of the definitions in this approach following the treatment of Eringen (1980 ▸, 1975 ▸).

### A1. Description of solid continua

In continuum mechanics a solid body *B* is assumed to have a continuous distribution of matter at all scales. This assumption implies that the constituent material is infinitely subdividable and, in a homogeneous body at the infinitesimal limit, all (approximately zero-dimensional) material volumes have identical physical properties. These ‘material points’ {*Q*′} are elements of the set constituting *B*. Each material point *Q*′ is associated with a geometric point *Q* which can be defined by its coordinates in a metric space, such as a coordinate manifold in Euclidian three space. Thus, the (geometric) point *Q* is the place occupied by the material point *Q*′. Neglecting rigid-body translations/rotations, the deformation of a continuum is characterized by the changes in the position of every material point in response to applied loads and/or tractions. In the literature, the coordinates for the deformed body are called spatial or Eulerian, whereas the coordinates in the un­deformed (natural) state are termed material or Lagrangian. These coordinates are used, respectively, to define Eulerian and Lagrangian strain tensors.

### A2. Definition of strain at a point

Consider the deformation/distortion of a continuum solid body by the application of external (surface) loads and/or tractions. If the displacement of any point within the body from its initial position is infinitesimally small, the geometry and material parameters (such as elastic moduli, density *etc.*) at each material point can be considered independent of deformation. Also, since for infinitesimal displacements the displacement gradient is much smaller than unity,  , it can be shown that the Lagrangian and Eulerian strain tensors **E*** and **e**, respectively, are approximately the same, and equal to the (infinitesimal) Cauchy strain tensor **ɛ** with components

Here ɛ_*ij*_ is a symmetric second-rank tensor and the comma between the subscripts indicates the partial derivative along the *x_j_* axis. Once ɛ_*ij*_ have been determined at a point *Q* for a given coordinate system *x*_*i*_, the strains  for any other coordinate system  can be obtained from the transformation law for second-rank tensors (Nye, 1985 ▸),

Here, *a*_*ij*_ are direction cosines linking the coordinate systems *x*_*i*_ and  , and summation over repeated indices (Einstein notation) is indicated.

We note that since, for infinitesimal displacements, **E*** ≃ **e** ≃ **ɛ** for all points *Q*, any differences in the material and spatial coordinates of a given material point in the continuum are negligible. Consequently, in what follows we will not distinguish spatial (geometric) and material points, and simply use ‘points’ at which all parameters of interest are defined. Note that a geometric point in a ‘continuum material’ is not equivalent to a geometric point in a crystalline solid. In the latter case the (geometric) point might fall on or between (Bohr) atoms, and the stress and strain definitions used here will not be applicable (Xiong *et al.*, 2019 ▸).

The components of the Cauchy strain tensor cannot take arbitrary values. Equation (16) defines a system of six differential equations for the specification of three displacement components *u*_*i*_. In order to ensure single-valued continuous displacement functions within the continuum (required to avoid gaps and/or overlaps of the material within the body), the (infinitesimal) strain terms ɛ_*ij*_ must satisfy the Saint-Venant compatibility equations (Eringen, 1980 ▸),

The compatibility condition can cause large differences in the strain state of a crystallite embedded in an aggregate compared with the same crystallite by itself, even if the far-field stresses are the same in both cases.

### A3. Definition of stress at a point

The total force in a cross section of the body, divided by the particular cross-sectional area, is termed mean (or nominal) stress. This parameter is of limited utility since, for the general loading case, internal forces (and moments) can vary from point to point. Consequently, local stresses defined at points must be used.

Consider a continuous body at static equilibrium with an arbitrary set of surface forces **F**. Within the entire body, internal contact forces and moments are transmitted from point to point such that local and global compatibility conditions are satisfied. Let *A* be an imaginary surface dividing the (continuous) body into two segments and containing the internal point *Q*. Using the Euler–Cauchy principle, the force distribution on an element of area Δ*A* containing *Q* with normal vector **n** is equivalent to a contact (surface) force Δ**F** exerted at *Q* and surface moment Δ**M**. Neglecting body forces, this contact force is given by

where **T** ^(**n**)^ is the mean surface traction.

Cauchy’s stress principle asserts that as Δ*A* tends to zero the surface moment Δ**M** vanishes and the mean surface traction **T** ^(**n**)^ tends to the stress vector  at the point *Q* associated with a plane with normal vector **n**,

Here **x**_*i*_ is the unit vector along Cartesian coordinate *x*_*i*_ (i = 1 to 3) of **n** and Δ*F_i_* is the contact force component along *x*_*i*_. Cauchy’s stress theorem states that at any point within the solid body there exists a symmetric second-rank tensor field, called the Cauchy stress tensor, such that **T** ^(**n**)^ is a linear function of **n**,

where σ_*ij*_ are the components of the (symmetric) Cauchy stress tensor (σ_*ij*_ = σ_*ji*_).

Equation (22) indicates that the stress vector **T** ^(**n**)^ at any point *P* in a solid continuum associated with a plane with normal unit vector **n** can be expressed as a function of the stress vectors on the planes perpendicular to the coordinate axes *x*_*i*_, *i.e.* in terms of the components σ_*ij*_ of the stress tensor **σ**. The components of the stress tensor  for any other coordinate system  can then be obtained from the transformation law for second-rank tensors,

For continuum bodies in static equilibrium the components of the Cauchy stress tensor must satisfy the conditions of equilibrium

at every point within the body and

at all points on the surface of the body.

### A4. Linear constitutive equations

In linear elasticity theory for homogeneous (continuum) solids, the strains induced in the body are linearly related to the stresses caused by applied loads/tractions through Hooke’s law (Nye, 1985 ▸),

where *C*_*ijkl*_ and *S*_*ijkl*_ are the stiffness and compliance tensors, respectively, of the material. Both *C*_*ijkl*_ and *S*_*ijkl*_ are fourth-rank symmetric tensors. Note that, while the forms of the stiffness and compliance tensors depend on the symmetry of the unit cell of a solid (if any), Cauchy stress and strain tensors and Hooke’s law cannot be applied directly to a single unit cell since a unit cell is not a continuum.

If the material is an isotropic continuum, with elastic properties independent of position or direction within the material (translational and rotational invariance, spherical symmetry), the *C*_*ijkl*_ and *S*_*ijkl*_ tensors have only two unique terms and the material has the same stiffness in all directions. In this case Hooke’s law [equations (25*a* [Disp-formula fd25a]) and (25*b* [Disp-formula fd25b])] is greatly simplified:

where *E* and ν denote Young’s modulus and Poisson’s ratio, respectively, of the (isotropic) material, and δ_*ij*_ is Kronecker’s delta.

In anisotropic single crystals some crystal directions in the solid can be much stiffer than others. For cubic crystals the effective Young modulus (stiffness) along any crystal direction [*hkl*] can be expressed as

Here, the compliance terms are referred to the unit-cell coordinate system along the 〈00*l*〉 axes. If the term in the brackets is zero, *i.e.* [(*S*_1111_ − *S*_1122_) − 2*S*_2323_] = 0, the stiffness of the cubic crystal is independent of crystal direction and the material exhibits an isotropic elastic response. This same term, re-arranged, is the Zener anisotropy index, *Z*_I_ = (*S*_1111_ − *S*_1122_)/2*S*_2323_.

## Appendix B Definition of free-body diagrams in Saint-Venant regions

When boundary effects are present, local stress/strain values may depend on position within the specimen, even for a microstructurally homogeneous material. An example is shown in Fig. 16 ▸, where isochromatic stress contours in a homogeneous acrylic cylinder placed under compression by a C-clamp are shown. This system of C-clamp and cylinder contains residual stresses; the compressive stresses in the cylinder are balanced by the stresses in the C-clamp. Regions *R*_1_ and *R*_3_ have Saint-Venant stresses and strains due to imperfections of the clamp surfaces, while region *R*_2_ has a homogeneous stress/strain distribution. Idealized free-body diagrams of these regions, each with two (example) ROIs *A* and *B*, are shown in Fig. 16 ▸(*b*). In region *R*_2_ the average stresses are equal to the local stresses; ROIs *A*_*R*2_ and *B*_*R*2_ possess identical stress states, which are equal to the far-field stresses at the boundaries of *R*_2_. This applies to any ROI of any size, including material points, within *R*_2_. For the Saint-Venant regions *R*_1_ and *R*_3_, the (average) stress state within any ROI depends on its position and its size.
